# Amyotrophic Lateral Sclerosis Is Accompanied by Protein Derangements in the Olfactory Bulb-Tract Axis

**DOI:** 10.3390/ijms21218311

**Published:** 2020-11-05

**Authors:** Mercedes Lachén-Montes, Naroa Mendizuri, Karina Ausin, Pol Andrés-Benito, Isidro Ferrer, Joaquín Fernández-Irigoyen, Enrique Santamaría

**Affiliations:** 1 Clinical Neuroproteomics Unit, Navarrabiomed, Complejo Hospitalario de Navarra (CHN), Universidad Pública de Navarra (UPNA), Irunlarrea 3, 31008 Pamplona, Spain; mercedes.lachen.montes@navarra.es (M.L.-M.); naroa.mendizuri.sanchez@navarra.es (N.M.); 2 Proteored-ISCIII, Proteomics Platform, Navarrabiomed, Complejo Hospitalario de Navarra (CHN), Universidad Pública de Navarra (UPNA), Irunlarrea 3, 31008 Pamplona, Spain; karina.ausin.perez@navarra.es; 3 IdiSNA, Navarra Institute for Health Research, 31008 Pamplona, Spain; 4 Bellvitge Biomedical Research Institute (IDIBELL), 08908 Hospitalet de Llobregat, Spain; pol.andres.benito@gmail.com (P.A.-B.); 8082ifa@gmail.com (I.F.); 5 CIBERNED (Network Centre of Biomedical Research of Neurodegenerative Diseases), Institute of Health Carlos III, 28031 Madrid, Spain; 6 Department of Pathology and Experimental Therapeutics, University of Barcelona, 08007 Hospitalet de Llobregat, Spain; 7 Institute of Neurosciences, University of Barcelona, 08007 Barcelona, Spain

**Keywords:** Amyotrophic lateral sclerosis, proteomics, signaling, olfactory bulb, olfactory tract, TDP-43 proteinopathy

## Abstract

Amyotrophic lateral sclerosis (ALS) is a fatal disease characterized by progressive muscle paralysis due to the degeneration of upper and lower motor neurons. Recent studies point out an involvement of the non-motor axis during disease progression. Despite smell impairment being considered a potential non-motor finding in ALS, the pathobiochemistry at the olfactory level remains unknown. Here, we applied an olfactory quantitative proteotyping approach to analyze the magnitude of the olfactory bulb (OB) proteostatic imbalance in ALS subjects (*n* = 12) with respect to controls (*n* = 8). Around 3% of the quantified OB proteome was differentially expressed, pinpointing aberrant protein expression involved in vesicle-mediated transport, macroautophagy, axon development and gliogenesis in ALS subjects. The overproduction of olfactory marker protein (*OMP*) points out an imbalance in the olfactory signal transduction in ALS. Accompanying the specific overexpression of glial fibrillary acidic protein (*GFAP*) and Bcl-xL in the olfactory tract (OT), a tangled disruption of signaling routes was evidenced across the OB–OT axis in ALS. In particular, the OB survival signaling dynamics clearly differ between ALS and frontotemporal lobar degeneration (FTLD), two faces of TDP-43 proteinopathy. To the best of our knowledge, this is the first report on high-throughput molecular characterization of the olfactory proteostasis in ALS.

## 1. Introduction

Amyotrophic lateral sclerosis (ALS) derives from a combined degeneration of upper and lower motor neurons in the spinal cord and motor cortex with a median survival rate of less than five years [1]. The prevalence is 3–5 cases per 100,000 inhabitants/year, affecting individuals of both genders with a peak incidence in ages across 50–65 years and showing a considerable phenotypic variability [2,3,4]. ALS may be categorized as sporadic ALS (sALS; 90% of cases) or familial ALS (fALS; 10% of the cases) which is linked to mutations in a large variety of apparently unrelated genes [5] although mutations in *SOD1*, *TARDBP*, *FUS* and *C9orf72* are collectively present in more than 50% of fALS cases [6]. The *TARDBP* gene encodes the TAR DNA-binding protein 43 (TDP43), one of the major components of inclusion bodies in motor neurons of ALS patients [7]. The clinical hallmark of ALS is the involvement of motor neurons which leads to muscle weakness and eventual paralysis. Despite all the progress made in the last decade, the etiopathogenesis of ALS is still unknown, and disease-modifying treatments that could slow ALS progression are very limited [3]. In addition to TDP43-protein aggregates, different mechanisms have been proposed to drive ALS pathogenesis such as impaired proteostasis, disturbed RNA metabolism, nucleocytoplasmic and cytoskeletal and axon-transports defects, impaired DNA repair, vesicle-transport defects, excitotoxicity, mitochondrial dysfunction, neuroinflammation, astrogliosis and oligodendrocyte dysfunction [8,9,10,11,12,13]. Despite the disruption of these mechanisms potentially interrelating with each other, triggering the degeneration and death of the motor neuron [14], it remains to be established whether these imbalances are involved in the pathogenic mechanism or are a secondary event of the ALS process.

Although primarily classified as a neuromuscular disease, novel neuropathological and imaging information indicate an involvement of the non-motor axis during ALS progression [3]. Therefore, the capacity to monitor extra-motor abnormalities could be useful to increase the diagnostic and prognostic potential in ALS. For instance, 50% of ALS patients present cognitive impairments related to frontotemporal dementia (FTD) [15,16]. In fact, it is well known that there is a common clinical spectrum between ALS and FTD [17,18]. Importantly, multiple studies point out that olfactory dysfunction may be a potential non-motor finding in both ALS and FTD, [19,20,21,22,23,24]. In addition, patient-derived olfactory mucosa has been proposed as a cellular model to define the non-neuronal contribution to ALS pathology [25]. TDP43-positive inclusions have been observed across secondary olfactory centers (orbitofrontal cortex and hippocampus), primary olfactory cortex and the olfactory bulb (OB) in post-mortem ALS brains [26]. Part of the human sensory system pathology is also recapitulated in SOD1G93A mice, the most commonly used mouse model of ALS which, however, presents no TDP43 inclusions [27,28,29]. However, the impact of ALS on olfactory structures remains to be elucidated. The olfactory system comprises a sensory organ, the olfactory epithelium (OE), located inside the nasal cavity together with the specific olfactory brain regions, which include the OB (the first brain region responsible for the processing of odor information) and the olfactory tract (OT). Thus, the scent’s molecular information is captured by the olfactory sensory neurons, located in the OE. Then, this information travels through the different layers of the OB, which include mitral and tufted cells, finally reaching the OT [30]. In view of clinical and neuropathological data, an in-depth molecular screening of the olfactory proteostasis is necessary to unveil the missing links in the biological understanding of the olfactory neurodegeneration in ALS. In this work, we applied an olfactory proteotyping approach based on quantitative mass-spectrometry and biochemical approaches to analyze the magnitude of the OB and OT proteome imbalance in the pathophysiology of ALS.

## 2. Results and Discussion

Taking into account that the severity of TDP-43 pathology follows a rostro-caudal gradient [31,32], previous neuroproteomic workflows have been mainly applied at cortical and spinal cord levels [33,34]. Being aware that ALS-associated proteins present cellular prion-like properties [35,36] and the OB is a site for prion-like propagation in neurological disorders [37], we have used OB proteomics [38,39,40] to characterize, for the first time, the potential disarrangement in the olfactory proteostasis that occur in ALS.

### 2.1. OB Proteome-Wide Analysis in Human ALS

**Figure 1:**
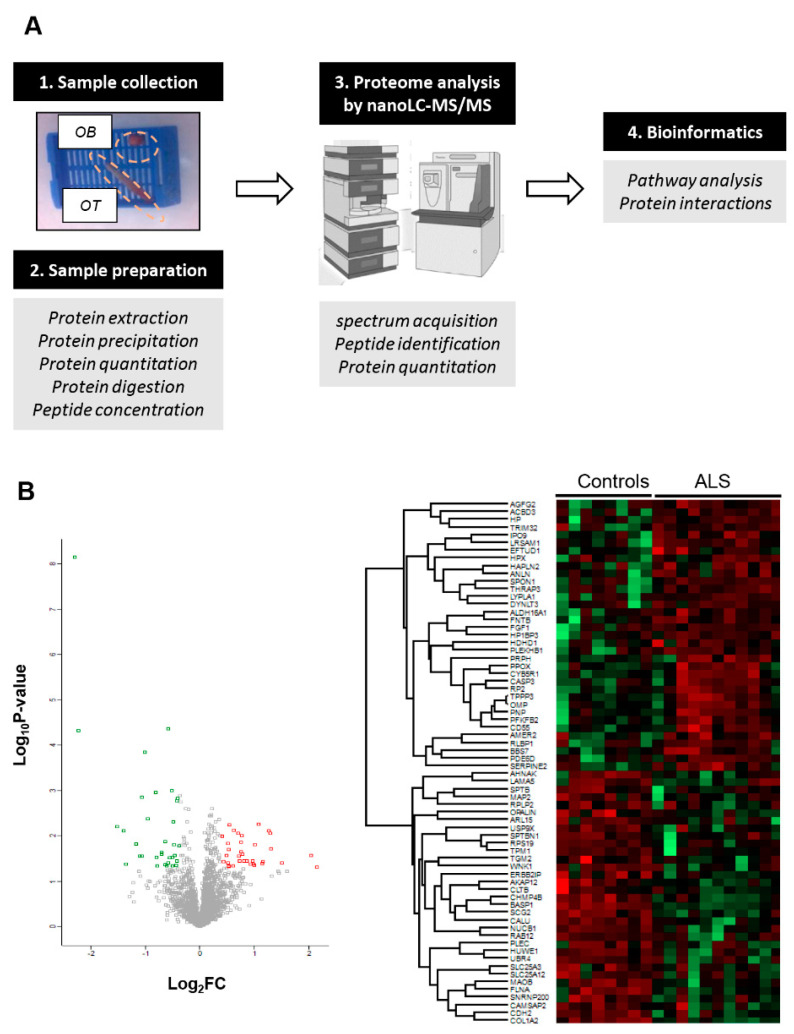
(**A**) An overview of the workflow used for the molecular characterization of the OB derived from ALS subjects (OB: olfactory bulb; OT: olfactory tract). (**B**) Volcano-plot representing the fold change of quantified proteins with associated *p*-value from the pair-wise quantitative comparisons of control vs ALS. Using international criteria, although 4777 proteins were identified, only proteins identified with at least two unique peptides were considered (2530 quantified proteins). A unique peptide is considered a peptide sequence that is exclusively present in a protein, and it is not shared across multiple proteins belonging for example to the same family. Down-regulated and up-regulated proteins are highlighted in green and red respectively (left). Heatmap representation showing both clustering and the degree of change for the differentially expressed proteins in ALS phenotype (Right).

An in-depth label-free quantitative proteomics approach (Figure 1A) was used to monitor the OB protein expression profiling derived from ALS cases (*n* = 12) and neurological intact controls (*n* = 8). Among 4777 identified proteins, 2530 proteins were quantified across all samples (Supplementary Table S1), from which 68 proteins were differentially expressed (DEPs) between both groups (35 upregulated and 33 downregulated proteins in ALS) (Figure 1B and Table 1).

**Figure 2:**
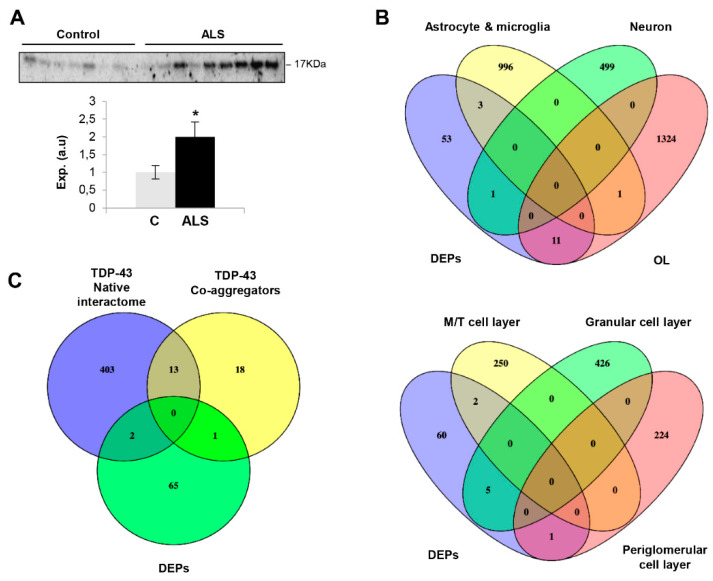
(**A**) OB Protein expression changes of OMP in ALS subjects by Western blotting. Data are presented as mean ± SEM. * *p* < 0.05 vs. control group; (a.u: arbitrary units). (**B**) Cluster-enriched genes in specific brain cell-types (upper) and OB cell layers (lower) that are differentially expressed at the level of the OB in ALS subjects. (**C**) Venn diagram showing the overlap between differential OB proteins and experimentally demonstrated TDP-43 interactors and co-aggregators. (DEPs: differential expressed proteins; OL: oligodendrocyte; M/T: mitral/tufted cells).

ALS-related proteins such as TDP43, SOD1 and FUS were unchanged in the OB (Supplementary Table S1). Curiously, 9 out of 68 DEPs have been previously linked to ALS (Table 1). Specifically, RPS19 is altered in reactive glial cells of hSOD1G93A mice [41]. *PLEC* and *PRPH* genes present mutations linked to familial ALS phenotypes [42,43]. MAP2 has been recently proposed as a CSF biomarker candidate in ALS [44]. MAOB and CASP3 activities are altered in the spinal cord of human ALS and motoneurons of SOD1-mutant mouse models, respectively [45,46]. SERPINE2 is a component of neurofilament conglomerates of motoneurons [47]. PLEKHB1 levels are deregulated in motoneurons at motor symptom onset in TDP-43-driven ALS models [48]. High CD55 levels have been observed in the motor end-plates of ALS [49]. Subsequent experiments were performed to check the levels of olfactory marker protein (OMP), an ALS-unrelated protein differentially expressed in ALS OBs. As shown in Figure 2A, OMP was upregulated in ALS at the level of the OB, confirming the mass-spectrometry results. OMP is involved in olfactory signal transduction with an apparent multitask role as a potential phosphodiesterase inhibitor upstream of cAMP production in olfactory sensory neuron (OSN) cilia [50], determining the duration of odor responses by OSNs [51,52], aiding the OSN maturation [53] and sparsening the primary olfactory input to the brain [54]. All this information together with the involvement of OMP in the formation and refinement of the olfactory glomerular map [55] points out that abnormal levels of OMP may trigger an aberrant interpretation of sensory stimuli in ALS patients.

It is important to note that the technological workflow applied is biased to the identification of highly abundant proteins, hampering the accurate detection and quantification of protein subsets expressed at low levels that may also be disrupted in ALS phenotypes. Moreover, our olfactory proteotyping is not able to differentiate between the OB cell layers. To overcome this drawback, previous single-cell RNA-seq datasets [56,57] were used to perform cell-type enrichment analysis across OB DEPs detected by mass-spectrometry. As shown in Figure 2B, a subset of olfactory DEPs is considered highly-enriched genes in specific brain cell populations: *SCG2* in neurons, *FGF1* in astrocytes, *TGM2* and *HP* in microglia and *COL1A2*, *OPALIN*, *SERPINE2*, *HAPLN2*, *TPPP3*, *PLEKHB1*, *ANLN*, *PDE6D*, *SPON1*, *RLBP1* and *OMP* in oligodendrocytes. Moreover, part of the proteostatic alterations tend to be specifically enriched in OB cell layers such as mitral/tufted cells (*SCGN2*, *SPON1*), periglomerullar cells (*SERPINE2*) and granular cell layers (*BASP1*, *TPM1*, *RPS19*, *MAP2*, *SPTBN1*) (Figure 2B). All these data help us to understand the molecular disturbances that accompany the neurodegenerative process across each cellular homeostasis in ALS.

### 2.2. Functional Analysis for the Differential Ob Proteome Detected in ALS

**Figure 3:**
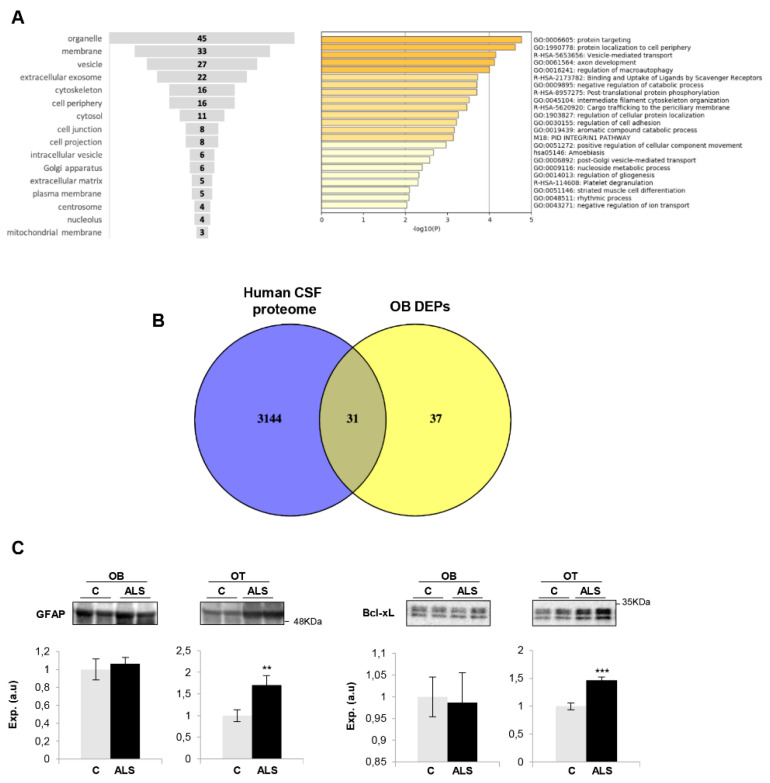
(**A**) Functional analysis of OB differentially expressed proteins in ALS. Cell-compartment distribution (left) and statistically significant enriched GO terms (right). (**B**) Venn diagram of proteins found in human CSF and differentially expressed proteins (DEPs) in ALS OBs. Numbers represent the number of shared proteins in the respective overlapping areas. (**C**) OT Protein expression changes of GFAP and Bcl-xL in ALS subjects by Western blotting. Data are presented as mean ± SEM. **p < 0.01 vs. control group; *** *p* < 0.001 vs. control group (a.u: arbitrary units). Equal loading of the gels was assessed by stain free digitalization.

Due to TDP43-positive inclusions having been observed in the OB from ALS subjects [26], we have interlocked the native and co-aggregating interactome from human TDP-43 [58] with the differential OB dataset to obtain additional information about the potential role of the OB aberrantly expressed proteins in the field of ALS. The downregulated RNA binding proteins SNRNP200 (U5 small nuclear ribonucleoprotein 200 kDa helicase) and AHNAK (neuroblast differentiation-associated protein AHNAK) are components of the native TDP-43 interactome (obtained from BIOGRID; https://thebiogrid.org/) (Figure 2C). PRPH (Peripherin), the most OB upregulated protein in ALS, is a co-aggregator of TDP-43 (Figure 2C), previously detected in motor neuron inclusions of ALS. According to cellular component analysis, DEPs were mainly mapped in membranes, organelles, vesicles and cytoskeleton (Figure 3A). A deep synaptic ontology analysis revealed that part of the protein derangements is directly related to the structural integrity of the synapse (Supplementary Table S2). To characterize the metabolic modulation at the level of the OB, the differential proteomic map (Table 1) was functionally analyzed. As shown in Figure 3A, vesicle-mediated transport (*p*-value: 71248E-05), macroautophagy (*p*-value: 99648E-05), nucleoside metabolism (*p*-value: 0.004), axon development (*p*-value: 766171E-05) and gliogenesis (*p*-value: 0.004) were part of the significantly overrepresented dysregulated processes in ALS subjects (Supplementary Table S3).

The cerebrospinal fluid (CSF) flows through the interstitial spaces of the brain, including a route through the olfactory system, along the lateral olfactory stria, down the olfactory tract (OT) to the OB [59]. Due to this olfactory CSF conduit, we consider that the application of olfactory proteomics [60,61] is an innovative approach to explore pathophysiological changes that might be monitored in CSF as a novel source of biomarkers in the context of ALS. For that, the OB differential dataset was compared with the most extensive human CSF proteome characterization [62]. As shown in Figure 3B, around 50% of the differential OB proteins have been previously detected in CSF, where 26 out of 31 proteins have not been previously linked to ALS (see Table 1), opening doors for these to be explored in biofluids as potential biomarkers for ALS diagnosis.

### 2.3. Imbalance in Survival Pathways Across the Olfactory Bulb-Tract Axis in ALS

**Figure 4:**
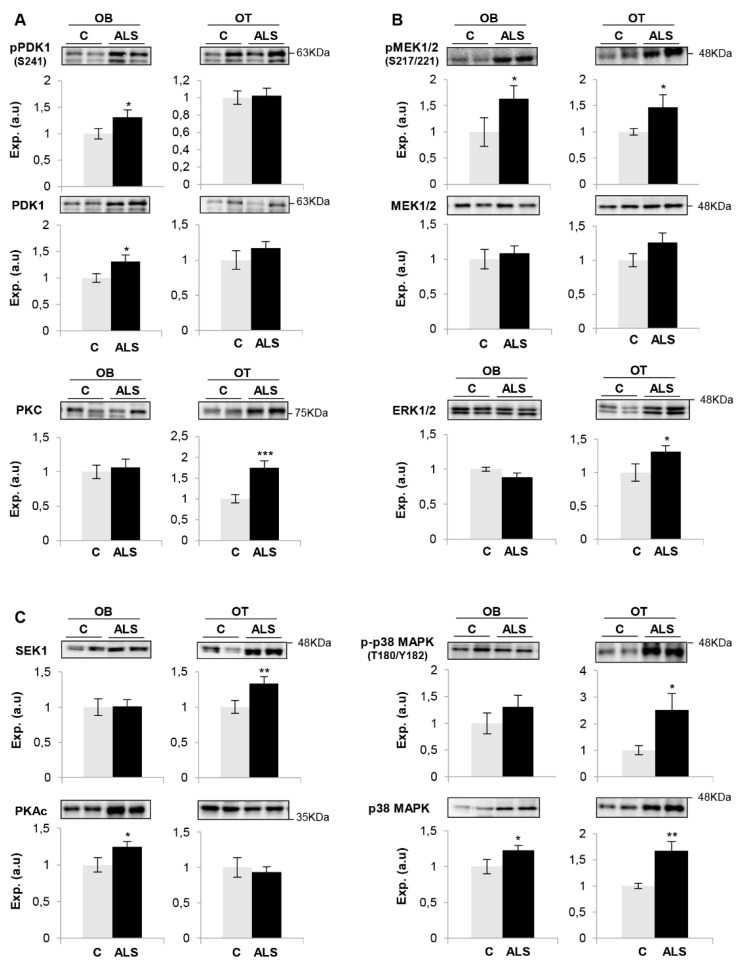
Signaling disruption across the OB-OT axis in ALS. State levels of PDK1, PKC (**A**), MEK1/2, ERK1/2 (**B**), SEK1, p38 MAPK, PKAc (**C**) across the OB-OT structures derived from controls and ALS subjects. Specific phosphorylation sites were also monitored in the case of PDK1, MEK1/2 and p38 MAPK. Data are presented as mean ± SEM. * *p* < 0.05 vs. control group; ** *p* < 0.01 vs. control group. Equal loading of the gels was assessed using stain-free imaging technology, and protein normalization was performed by measuring total protein directly on the gels (a.u; arbitrary units).

Much effort has been spent on studying the role of TDP-43 in human ALS pathogenesis but the available information is insufficient to understand the neurodegenerative impact at the level of olfactory areas. The olfactory tract (OT) is constituted by the axons coming from the mitral and tufted neurons located in the OB. Considering that the OT analysis may provide additional clues about the molecular disruptions along the olfactory system during the neurodegenerative process, subsequent experiments were performed to monitor olfactory astrogliosis across the OB–OT axis in ALS. We observed a specific increase in glial fibrillary acidic protein (GFAP) in parallel with an overproduction of Bcl-xL in ALS OTs (Figure 3C). Both molecules are correlatively elevated in ALS (G93A) mice and affected humans, being considered relevant mediators in the astrocytic response to oxidative stress in ALS [63]. It is well known that multiple protein kinases are linked to a plethora of pathological mechanisms present in ALS and are considered to be the major drug target family exploited in the last decades [64]. Moreover, previous studies from our group have demonstrated a differential protein kinase imbalance across tauopathies, sinucleinopathies and tardopathies at the olfactory level [39,40,65]. Subsequent experiments were performed to partially monitor the signaling dynamics present in the OB-OT axis derived from ALS subjects. As shown in Figure 4, a significant increase in steady-state levels of PDK1 and PKAc was exclusively observed in ALS OBs with respect to controls (Figure 4A,C). While this is the first report linking PDK1 with ALS, it has been demonstrated that synaptic restoration by PKA drives activity-dependent neuroprotection to motoneurons in a mutSOD1 ALS mouse model [66]. On the other hand, steady-state levels of PKC, SEK1 and ERK1/2 were significantly increased at the level of OT in ALS subjects (Figure 4A–C). PKC activity is increased in ALS patients, suggesting effects on neuronal viability through voltage-dependent calcium channel regulation [67]. Moreover, several PKC isoforms are regulated and localized in the pre- and postsynaptic zones in the neuromuscular junctions, affected in ALS muscles [68,69]. Due to the complex functionality covered by all PKC isoforms in neurite outgrowth and synaptic plasticity, further exploration is needed to decipher the specific role of each PKC isoform (and its post-translational modifications) during the neurodegenerative process in ALS. We observed an overexpression of OT SEK1, an upstream regulator of JNK/SAPK. This pathway may play a neuroprotective role in motor neurons in ALS [70]; however, this route was unchanged across the ALS OB–OT axis (Supplementary Materials). ERK1/2 has been associated with TDP-43 aggregation [71]. Moreover, a significant increase in the activation state of MEK1/2 as well as an overexpression of p38 MAPK was observed across the OB–OT axis in ALS (Figure 4B,C). Interestingly, different therapeutic approaches leading to inhibit MEK1/2 inhibition have been established for potential ALS treatments. PD98059 reduces the phosphorylation of neurofilaments [72] and trametinib has shown preclinical efficacy in ALS models (ClinicalTrials.gov Identifier NCT04326283). On the other hand, neurotoxic effects due to the appearance of reactive glial cells and the hyperphosphorylation of neurofilaments have been attributed to overexpression of p38 MAPK in the spinal motoneurons of transgenic SOD1 mice, increasing the production of nitric oxide and the apoptosis activation [73]. No appreciable changes were observed in the activation status of AKT, SAPK/JNK and CaMKII across the OB–OT axis in ALS (Supplementary Materials). Our data indicate that ALS induces a differential and tangled disruption in specific olfactory survival pathways, compromising the phosphorylation state and potentially the activity of multiple substrates at the level of the OB and OT, contributing to the increase of the proteostatic imbalance present at advanced stages of the disease.

On the whole, the kinase expression and/or activation profile partially differs between spinal cord and olfactory areas in ALS [74]. However, a downregulation in CaMKII, MEK1/2, PDK1 and PKC protein levels have been evidenced in frontotemporal lobar degeneration (FTLD)-TDP43 subjects in respect to controls, maintaining normal levels of AKT, p38 MAPK and PKAc at the level of the OB [65]. All these data suggest that despite ALS and FTLD being two faces of TDP-43 proteinopathy with an overlap in clinical presentation and neuropathology, the OB proteostatic alterations clearly differ between both phenotypes. However, only 1–3% of the quantified OB proteome is aberrantly expressed in FTLD and ALS, compared with the olfactory proteostatic imbalance previously characterized in Alzheimer’s and Parkinson’s diseases (around 20%) [39,40]. Bearing in mind that additional studies are needed to evaluate the olfactory activation state of kinases responsible for TDP-43 phosphorylation/aggregation (CK-1δ, TTBK1/2 or CDC7) [64], our results lay the foundation for future exhaustive phosphoproteomics studies across the OB–OT axis in order to increase our understanding about the pathobiology that accompanies the neurodegenerative process across TDP-43 proteinopathies.

## 3. Materials and Methods

The workflow followed in this study is summarized in Figure 1A.

### 3.1. Materials

The following reagents and materials were used: anti-Bcl-xL (ref. 2764), anti-pAkt (Ser473) (ref. 4060), anti-Akt (ref. 4685), anti-pMEK1/2 (Ser217/221) (ref. 9154), anti-MEK1/2 (ref. 9126), anti-pERK1/2 (Thr202/Tyr204) (ref. 4370), anti-ERK1/2 (ref. 9102), anti-pPKA (Thr197) (ref. 5661), anti-PKA (ref. 4782), anti-pSEK1/MKK4 (Ser257/Thr261) (ref. 9156), anti-SEK1/MKK4 (ref. 9152), anti pSAPK/JNK (Thr183/Tyr185) (ref. 9255), anti pSAPK/JNK (ref. 9252S), anti p-p38 MAPK (Thr180/Tyr182) (ref. 4511), anti p38 MAPK (ref. 9212), anti-pCAMKII (Thr286) (ref. 12,716), anti-CAMKII (ref.11945), anti-pPDK1 (ser241) (ref. 3061), anti-PDK1 (ref. 3062) and anti-pPKC-pan (ref. 9379) were purchased from Cell signaling. Anti-GFAP (ref. ab7260) was purchased from Abcam. Anti PKC-pan (ref. SAB4502356) was from Sigma Aldrich. Electrophoresis reagents were purchased from Biorad and trypsin from Promega.

### 3.2. Human Samples

According to the Spanish Law 14/2007 of Biomedical Research, informed written consent forms of the Brain Bank of IDIBELL (Barcelona, Spain) were obtained for research purposes from relatives of patients included in this study. Post-mortem fresh-frozen olfactory bulbs and tracts of 12 ALS patients, and 8 age- and gender-matched controls, were obtained from the Brain Bank of IDIBELL (Barcelona, Spain) following the guidelines of Spanish legislation. The control group is composed of elderly subjects with no histological findings of any neurological disease. The study was conducted in accordance with the Declaration of Helsinki and all assessments, post-mortem evaluations, and procedures were previously approved by the Local Clinical Ethics Committee (PI_2019/108). All human brains considered in the proteomics and follow-up phases had a post-mortem interval (PMI) lower than 19 h (Table 2). In all cases, neuropathological assessment was performed according to standardized neuropathological guidelines [75].

### 3.3. Sample Preparation for Proteomic Analysis

Whole OB specimens (70–80 mg) derived from controls and ALS cases were homogenized using ‘’mini potters’’ in lysis buffer containing 7 M urea, 2 M thiourea and 50 mM DTT. The homogenates were spun down at 100,000× *g* for 1 h at 15 °C. Before proteomic analysis, protein extracts were precipitated and pellets were dissolved in 6 M Urea and Tris 100 mM pH 7.8. Protein quantitation was performed with the Bradford assay kit (Bio-Rad). The protein extract for each sample was diluted in Laemmli sample buffer and loaded into a 0.75-mm-thick polyacrylamide gel with a 4% stacking gel casted over a 12.5% resolving gel. The run was stopped as soon as the front entered 3 mm into the resolving gel so that the whole proteome became concentrated in the stacking/resolving gel interface. Bands were stained with Coomassie Brilliant Blue and excised from the gel. Protein enzymatic cleavage (10 ug) was carried out with trypsin (Promega; 1:20, w/w) at 37 °C for 16 h as previously described (Shevchenko et al., 2006). Peptides were purified and concentrated using C18 Zip Tip Solid Phase Extraction (Millipore, Burlington, MA, USA).

### 3.4. Label Free LC-MS/MS

Mass spectrometric analyses were performed on an EASY-nLC 1200 liquid chromatography system interfaced with a Q Exactive HF-X mass spectrometer (Thermo Scientific) via a nanospray flex ion source. A total of 1 ug of tryptic peptides was loaded onto an Acclaim PepMap100 precolumn (75 um × 2 cm, Thermo Scientific) connected to an Acclaim PepMap RSLC (75 um × 25 cm, Thermo Scientific) analytical column. Peptides were eluted from the column at a flow rate of 250 nL min-1 using the following percentage of buffer B in the gradient (buffer A: 0.1% formic acid, buffer B: 80% acetonitrile, 0.1% formic acid): 3 min 5%, 7 min 5–7%, 105 min 7–22%, 25 min 22–27%, 15 min 27–31%, 28 min 31–53%, 1 min 53–95%. The mass spectrometer was operated in positive ion mode and a data-dependent acquisition mode was used. The spray voltage was set at 1.9 keV, the capillary temperature at 300 °C and the S-Lens RF level at 50. Full MS scans were acquired from m/z 375 to 1800 with a resolution of 60,000 at m/z 200. The 15 most intense ions were fragmented by higher energy C-trap dissociation with normalized collision energy of 28 and MS/MS spectra were recorded with a resolution of 15,000 at m/z 200. The maximum ion injection time was 45 ms for survey and for MS/MS scans, whereas AGC target values of 3 × 106 and 5 × 105 were used for survey and MS/MS scans, respectively. A dynamic exclusion time of 40 s was applied and singly charged ions, ions with 6 or more charges, and ions with unassigned charge states were excluded from MS/MS. Data were acquired using Xcalibur software (Thermo Scientific).

### 3.5. Protein Identification and Quantification

Mass spectrometry raw data were processed using the MaxQuant software (v.1.6.3.3) [76] following the next parameters. Data were searched against the Homo Sapiens UniProtKB database (February 2019) also containing frequent contaminants and the reversed version of all sequences. In the group-specific parameters, main peptide search and first search tolerance were set as 4.5 ppm and 20 ppm, respectively. In addition, trypsin was selected as the enzyme with a maximum of two missed cleavages, and methionine oxidation and N-terminal acetylation were selected as variable modifications whereas carbamidomethylation was selected as fixed. In the global parameters, the minimum peptide length was set to 7 amino acids, fragment mass deviation to 40 ppm and false discovery rate (FDR) for peptide spectrum match (PSM) and peptide and protein identification were set to 1%. The output file ‘’proteingroups.txt’’ generated by MaxQuant was then analyzed with the Perseus software (v.1.6.2.3) [77]. Potential contaminants and proteins identified as reversed were removed. Protein identification was considered valid with at least two unique or razor peptides whereas protein quantification was calculated using at least two unique peptides. Statistical significance was calculated by a two-way Student’s t-test (*p* < 0.05) and a 1.3-fold change cut-off was used. Thus, proteins with ratios below the low range of 0.77 were considered downregulated whereas those above the high range 1.33 were considered upregulated. Data visualization was also performed with Perseus. MS raw data and search results files were deposited in the Proteome Xchange Consortium (http://proteomecentral.proteomexchange.org) via the PRIDE partner repository [78] with the identifier PXD021630 (reviewer account details: Username: reviewer_pxd021630@ebi.ac.uk; Password: akmkhVDw).

### 3.6. Bioinformatics

TDP43 interactome and pathway analysis were performed by BioGrid [79] and Metascape [80] respectively.

### 3.7. Western-Blotting

Equal amounts of protein (10 µg) were resolved in 4–15% stain free SDS-PAGE gels (Bio-rad). OB and OT proteins derived from control and ALS subjects (Table 2) were electrophoretically transferred onto nitrocellulose membranes using a Trans-blot Turbo transfer system (up to 25 V, 7 min) (Bio-rad). Membranes were probed with primary antibodies (between 1:200 and 1:1000 dilution) in 5% nonfat milk or BSA according to manufacturer instructions. After incubation with the appropriate horseradish peroxidase-conjugated secondary antibody (1:5000), the immunoreactivity was visualized by enhanced chemiluminiscence (Perkin Elmer) and detected by a Chemidoc MP Imaging System (Bio-Rad). Equal loading of the gels was assessed by stain free digitalization. After densitometric analyses (Image Lab Software Version 5.2; Bio-Rad), optical density values were normalized to total stain in each gel lane and expressed as arbitrary units.

## 4. Conclusions

Our work provides novel insights regarding the olfactory proteostatic imbalance present in ALS. Besides the pathological TDP-43 depositions previously observed in olfactory areas, we have unveiled OB proteostatic rearrangements (only 3% of the quantified OB proteome), mainly affecting vesicle-mediated transport, macroautophagy, and axon development as well as cell survival routes. Nine differentially expressed proteins have been previously linked to ALS (RPS19, *PLEC*, *PRPH,* MAP2, MAOB, CASP3, SERPINE2, PLEKHB1, CD55). Interestingly, the imbalance in OMP protein levels together with the disruption in PDK1/PKC, MEK/ERK, SEK1 and the p38 MAPK axis suggest a partial alteration in the odor signal transduction across the OB–OT axis in ALS. Our data facilitate the understanding of the role of the olfactory axis in ALS pathophysiology, identifying not only differences between TDP-43 proteinopathies but also 26 ALS-unrelated protein intermediates that may be explored in CSF as candidate biomarkers for ALS diagnosis in early stages of the disease.

## Figures and Tables

**Table 1:** Significantly deregulated proteins in OB derived from ALS subjects.

Protein ID	Protein Name	Gene	*p* Value	FC	Activity/Pathway
P80723	Brain acid soluble protein 1	BASP1	0.00	0.20	transcription corepressor activity
P08123	Collagen alpha-2(I) chain	COL1A2	0.00	0.21	Integrin Pathway & Collagen trimerization
Q96PE5	Opalin	OPALIN	0.01	0.35	oligodendrocyte terminal differentiation
Q7Z6Z7	E3 ubiquitin-protein ligase HUWE1	HUWE1	0.01	0.38	proteasomal degradation
O15230	Laminin subunit alpha-5	LAMA5	0.04	0.39	Integrin Pathway & signaling by GPCR
Q09666	Neuroblast differentiation-associated protein AHNAK	AHNAK	0.02	0.44	Phospholipase-C Pathway
F5H7S3	Tropomyosin alpha-1 chain	TPM1	0.03	0.47	cytoskeletal protein binding
O43852	Calumenin	CALU	0.00	0.48	calcium ion binding
Q5T4S7	E3 ubiquitin-protein ligase UBR4	UBR4	0.03	0.48	ubiquitin-protein transferase activity
P13521	Secretogranin-2	SCG2	0.00	0.50	chemoattractant activity
Q02818	Nucleobindin-1	NUCB1	0.00	0.52	Golgi calcium homeostasis
P21333	Filamin-A	FLNA	0.00	0.57	crosslink actin filaments
P39019	40S ribosomal protein S19	RPS19	0.03	0.58	pre-rRNA processing
Q6IQ22	Ras-related protein Rab-12	RAB12	0.05	0.58	Vesicle trafficking
O75643	U5 small nuclear ribonucleoprotein 200 kDa helicase	SNRNP200	0.02	0.61	mRNA splicing
P11277	Spectrin beta chain, erythrocytic	SPTB	0.03	0.62	actin filament binding
Q93008	Probable ubiquitin carboxyl-terminal hydrolase FAF-X	USP9X	0.01	0.64	deubiquitinase, protein turnover
Q9NXU5	ADP-ribosylation factor-like protein 15	ARL15	0.04	0.64	GTP binding
Q15149	Plectin	PLEC	0.05	0.66	Cytoskeleton remodeling Neurofilaments
F5GWT4	Serine/threonine-protein kinase WNK1	WNK1	0.04	0.67	regulation of electrolyte homeostasis
Q9H444	Charged multivesicular body protein 4b	CHMP4B	0.00	0.67	sorting of endocytosed cell-surface receptors
Q08AD1	Calmodulin-regulated spectrin-associated protein 2	CAMSAP2	0.03	0.68	regulator of neuronal polarity
Q02952	A-kinase anchor protein 12	AKAP12	0.00	0.70	subcellular compartmentation of PKA/PKC
P21980	Protein-glutamine gamma-glutamyltransferase 2	TGM2	0.03	0.71	cross-linking and conjugation of polyamines
P11137	Microtubule-associated protein 2	MAP2	0.04	0.71	stabilization of microtubules
Q96RT1	Protein LAP2	ERBB2IP	0.00	0.72	Inhibits proinflammatory cytokine secretion
Q01082	Spectrin beta chain, non-erythrocytic 1	SPTBN1	0.02	0.72	movement of the cytoskeleton
P05387	60S acidic ribosomal protein P2	RPLP2	0.03	0.73	protein synthesis
O75746	Calcium-binding mitochondrial carrier protein Aralar1	SLC25A12	0.04	0.74	exchange of Asp for Glu in the mitochondria
P27338	Amine oxidase [flavin-containing] B	MAOB	0.00	0.75	metabolism of neuroactive and vasoactive amines
Q00325	Phosphate carrier protein, mitochondrial	SLC25A3	0.04	0.75	regulation of mitochondrial permeability
P19022	Cadherin-2	CDH2	0.00	0.75	cell-cell adhesion
P09497	Clathrin light chain B	CLTB	0.02	0.77	vesicle biogenesis
P07093	Glia-derived nexin	SERPINE2	0.01	1.33	endopeptidase inhibitor activity
Q08623	Pseudouridine-5-phosphatase	HDHD1	0.04	1.35	pyrimidine nucleoside salvage
Q7Z2Z2	Elongation factor Tu GTP-binding domain-containing protein 1	EFTUD1	0.03	1.41	translational activation of ribosomes
P00491	Purine nucleoside phosphorylase	PNP	0.01	1.43	nucleoside binding
P49356	Protein farnesyltransferase subunit beta	FNTB	0.01	1.43	farnesyltransferase activity
O60825	6-phosphofructo-2-kinase/fructose-2,6-bisphosphatase 2	PFKFB2	0.04	1.44	Synthesis/degradation of fructose 2,6-bisP
Q9H3P7	Golgi resident protein GCP60	ACBD3	0.02	1.44	maintenance of Golgi structure
P50336	Protoporphyrinogen oxidase	PPOX	0.05	1.45	heme biosynthesis
Q9GZV7	Hyaluronan and proteoglycan link protein 2	HAPLN2	0.05	1.45	establishment of blood-nerve barrier
Q96P70	Importin-9	IPO9	0.01	1.46	nuclear protein import
P02790	Hemopexin	HPX	0.04	1.53	heme transport
Q9BW30	Tubulin polymerization-promoting protein family member 3	TPPP3	0.01	1.54	Regulator of microtubule dynamics
Q9UHQ9	NADH-cytochrome b5 reductase 1	CYB5R1	0.01	1.62	desaturation/elongation of fatty acids
O75608	Acyl-protein thioesterase 1	LYPLA1	0.03	1.64	phospholipase activity
Q5SSJ5	Heterochromatin protein 1-binding protein 3	HP1BP3	0.04	1.66	heterochromatin organization
Q13049	E3 ubiquitin-protein ligase TRIM32	TRIM32	0.02	1.69	ubiquitin-protein transferase activity
O95081	Arf-GAP domain and FG repeat-containing protein 2	AGFG2	0.01	1.70	GTPase activator activity
Q8IWZ6	Bardet-Biedl syndrome 7 protein	BBS7	0.01	1.70	cilium assembly
F5GZH3	Pleckstrin homology domain-containing family B member 1	PLEKHB1	0.03	1.73	cell differentiation
A6NGJ0	Dynein light chain Tctex-type 3	DYNLT3	0.04	1.76	intracellular retrograde motility of vesicles
Q8IZ83	Aldehyde dehydrogenase family 16 member A1	ALDH16A1	0.04	1.82	oxidoreductase activity
Q9NQW6	Actin-binding protein anillin	ANLN	0.04	1.91	actomyosin contractile ring assembly
P05230	Fibroblast growth factor 1	FGF1	0.04	1.96	Integrin binding
O43924	GMP-Phosphodiesterase delta	PDE6D	0.04	1.97	ciliary targeting of farnesylated proteins
P42574	Caspase-3;Caspase-3 subunit p17;Caspase-3 subunit p12	CASP3	0.04	1.99	apoptosis execution
Q9HCB6	Spondin-1	SPON1	0.02	2.01	attachment of spinal cord & sensory neuron cells
Q6UWE0	E3 ubiquitin-protein ligase LRSAM1	LRSAM1	0.01	2.12	ubiquitin-protein transferase activity
P12271	Retinaldehyde-binding protein 1	RLBP1	0.04	2.22	retinoid metabolism
Q8N7J2	APC membrane recruitment protein 2	AMER2	0.04	2.23	Wnt signaling pathway
O75695	Protein XRP2	RP2	0.01	2.40	post-Golgi vesicle-mediated transport
H3BLV0	Complement decay-accelerating factor	CD55	0.01	2.45	regulation of the complement cascade
Q9Y2W1	Thyroid hormone receptor-associated protein 3	THRAP3	0.02	2.49	regulation of mRNA splicing & transcription
P47874	Olfactory marker protein	OMP	0.04	2.84	modulator of the olfactory signal-transduction
P00738	Haptoglobin	HPR	0.03	4.12	antioxidant activity
P41219	Peripherin	PRPH	0.05	4.46	Class-III neuronal intermediate filament

**Table 2:** Clinicopathological data of ALS subjects included in this study.

Groups	Age (years)	Onset	Sex	PMI	Neuropathological Diagnosis	AD Stages	OB Analysis	OT Analysis
**Control**	65	-	F	3 h 45 m	Status cribosus	I/0	Yes	No
	74	-	M	9 h 25 m	Lacunar infarction	III/A	Yes	No
	45	-	M	18 h 30 m	Status cribosus	0/0	Yes	No
	51	-	F	4 h	No lesions	0/0	Yes	Yes
	67	-	M	5 h 50 m	Amyloid angiopathy	I/0	Yes	Yes
	59	-	F	5 h 30 m	Metastatic carcinoma	I/0	Yes	Yes
	60	-	F	12 h	Status cribosus	I/0	Yes	Yes
	75	-	M	5 h 30 m	Status cribosus	I/0	Yes	Yes
**ALS**	57	Bulbar	M	4 h	ALS	IIA	Yes	Yes
	75	Bulbar	F	4 h 5 m	ALS	IIA	Yes	Yes
	79	Spinal	F	2 h 10 m	ALS	IIA	Yes	Yes
	57	Bulbar	F	10 h	ALS	I/0	Yes	Yes
	50	Spinal	M	10 h 10 m	ALS	I/0	Yes	Yes
	75	Bulbar	M	3 h	ALS	II/B	Yes	Yes
	71	Spinal	M	3 h 25 m	ALS	I/0	Yes	Yes
	68	Bulbar	F	16 h 30 m	ALS	I/0	Yes	Yes
	63	Spinal	F	18 h	ALS	I/0	Yes	Yes
	53	Bulbar	F	10 h	ALS	0/0	Yes	Yes
	71	Bulbar	F	18 h	ALS	I/A	Yes	Yes
	45	Spinal	F	4 h	ALS	0/0	Yes	Yes

## Supplementary Materials

Supplementary materials can be found at https://www.mdpi.com/1422-0067/21/21/8311/s1. Supplementary material includes additional loading controls and Western-blots; Table S1: Quantified OB proteome; Table S2: Synaptic Ontology; Table S3: Functional analysis.

## Abbreviations

ALS	Amyotrophic lateral sclerosis
CAMK II	Calmodulin-dependent protein kinase II
ERK	Extracellular signal-regulated kinase
FDR	False discovery rate
MEK	Mitogen-activated protein kinase kinase
OB/OT	Olfactory bulb/olfactory tract
OMP	Olfactory marker protein
p38 MAPK	p38 mitogen-activated protein kinase
PKA	Protein kinase A
PKC	Protein kinase C
PMI	Post-mortem interval
SAPK/JNK	(stress-activated protein kinase/Jun-amino terminal kinase)
SEK1	Mitogen-activated protein kinase Kinase 4
TDP43	TAR DNA-binding protein 43
