# Supplementary material for: Amyotrophic Lateral Sclerosis Is Accompanied by Protein Derangements in the Olfactory Bulb-Tract Axis
Source: Int J Mol Sci. 2020 Nov 5;21(21):8311. doi: 10.3390/ijms21218311 (PMC7664257; doi:10.3390/ijms21218311)
Supplement: Supplementary file 1 [file ijms-21-08311-s001.zip › supplementary files/SUPPLEMENTARY MATERIAL WB ALS.pptx]

## Slide 1
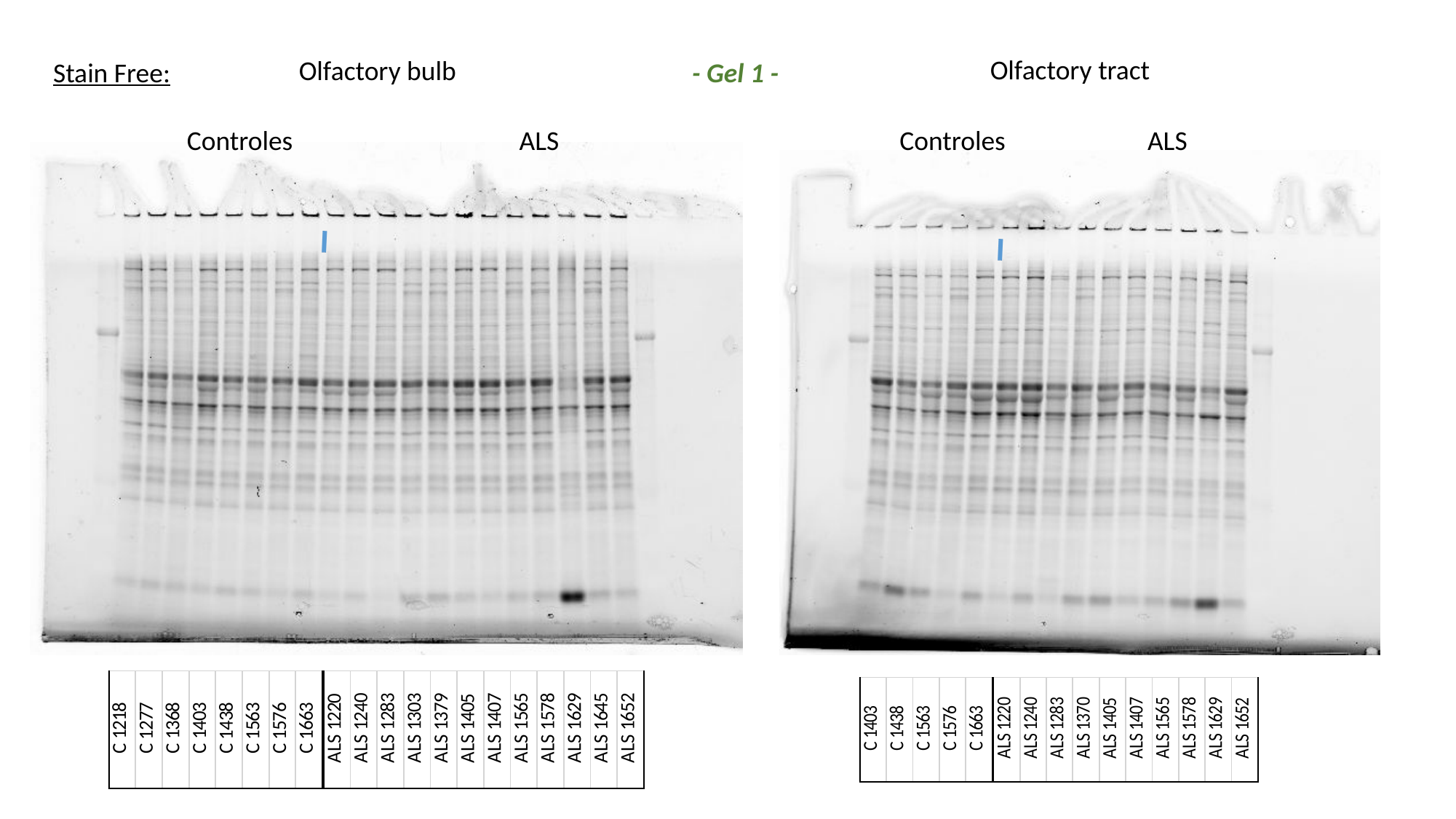

Olfactory tract
Olfactory bulb
Stain Free:
- Gel 1 -
Controles
Controles
ALS
ALS

## Slide 2
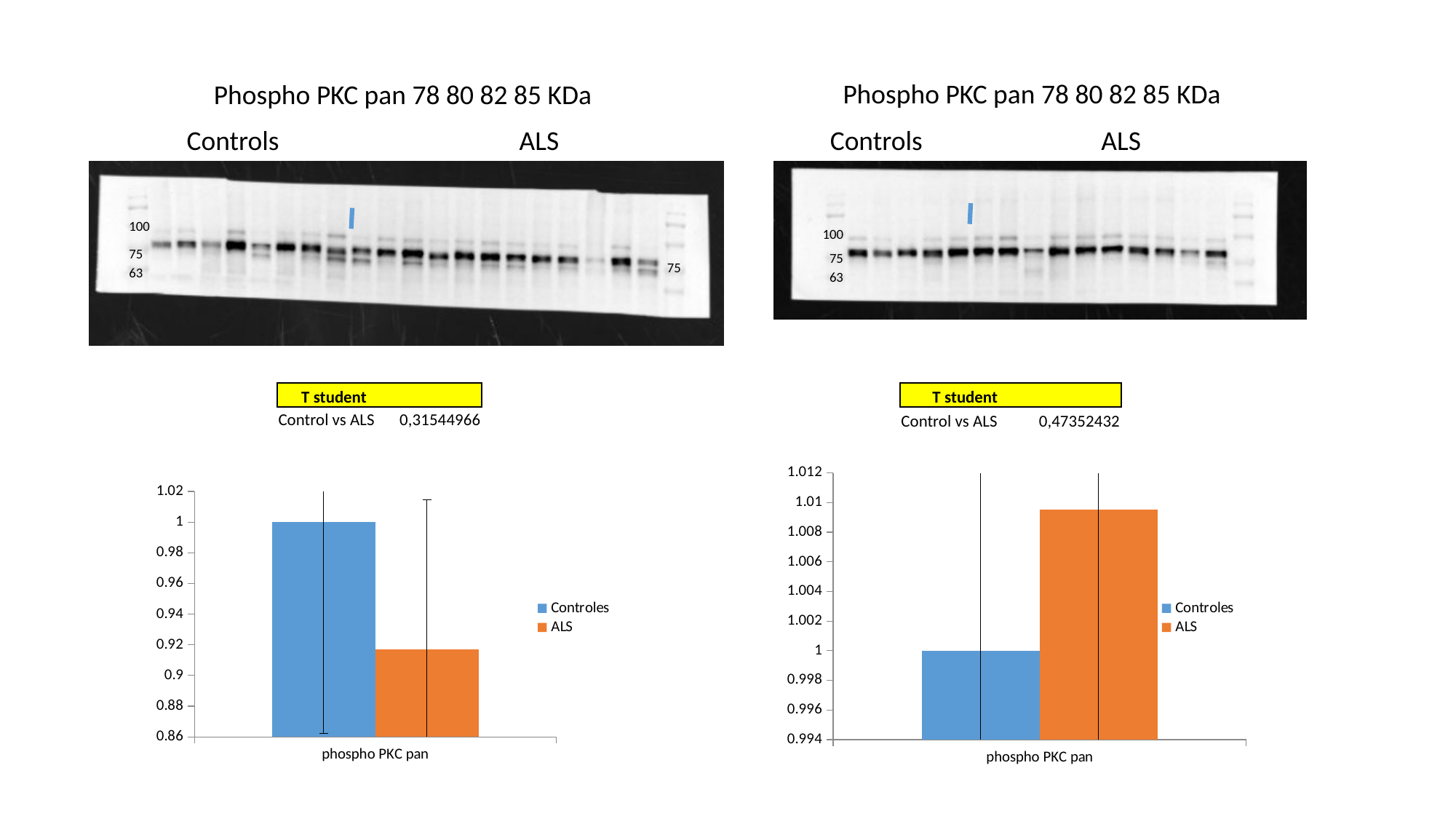

Phospho PKC pan 78 80 82 85 KDa
Phospho PKC pan 78 80 82 85 KDa
Controls
Controls
ALS
ALS
100
100
75
75
75
63
63
| T student | |
| --- | --- |
| Control vs ALS | 0,31544966 |
| T student | |
| --- | --- |
| Control vs ALS | 0,47352432 |
### Chart
| Category | Controles | ALS |
|---|---|---|
| phospho PKC pan | 1.0 | 1.0095405046504378 |
### Chart
| Category | Controles | ALS |
|---|---|---|
| phospho PKC pan | 1.0 | 0.9171009022596323 |

## Slide 3
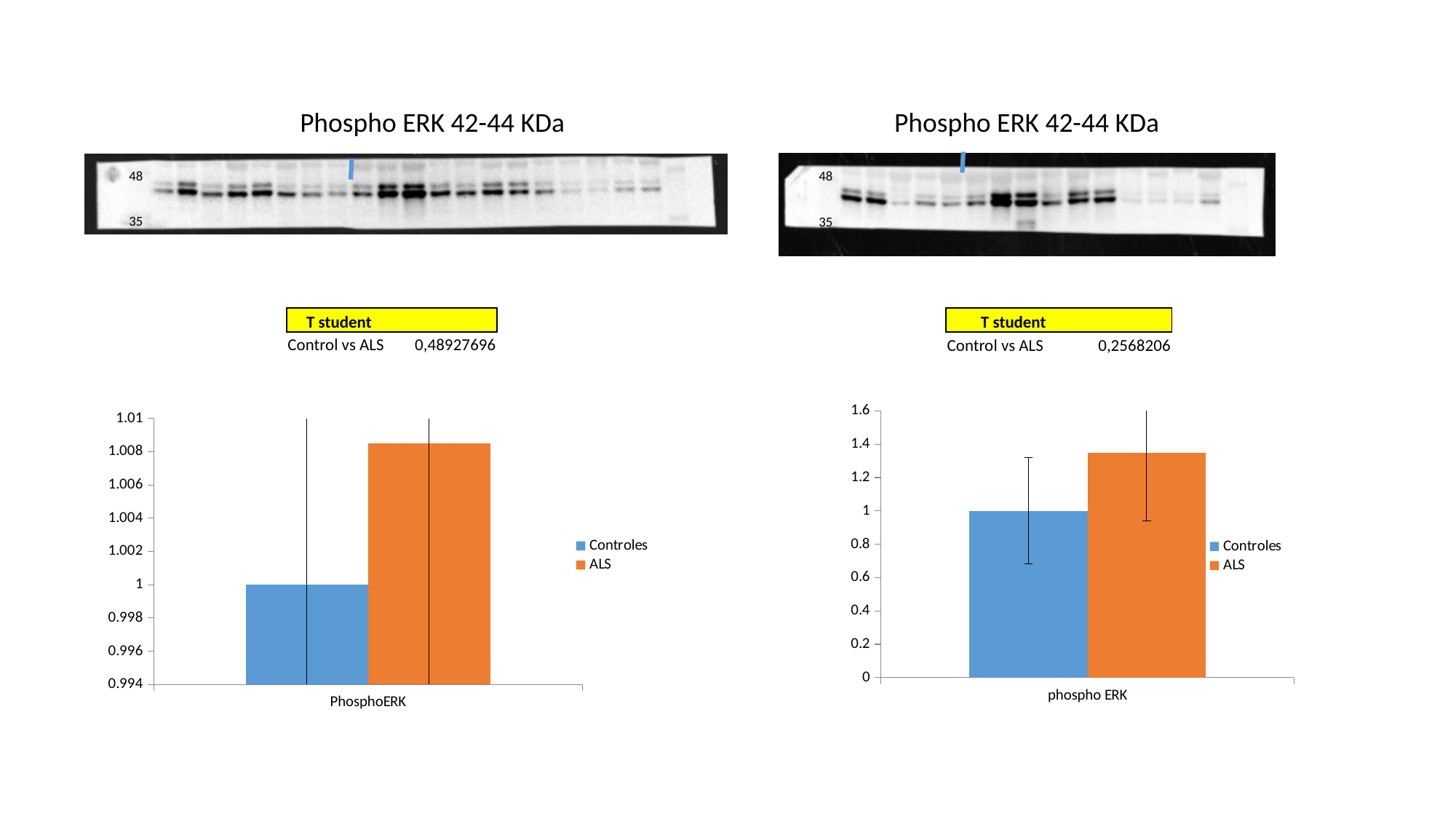

Phospho ERK 42-44 KDa
Phospho ERK 42-44 KDa
48
48
35
35
| T student | |
| --- | --- |
| Control vs ALS | 0,48927696 |
| T student | |
| --- | --- |
| Control vs ALS | 0,2568206 |
### Chart
| Category | Controles | ALS |
|---|---|---|
| PhosphoERK | 1.0 | 1.008496965617484 |
### Chart
| Category | Controles | ALS |
|---|---|---|
| phospho ERK | 1.0 | 1.3467700194491565 |

## Slide 4
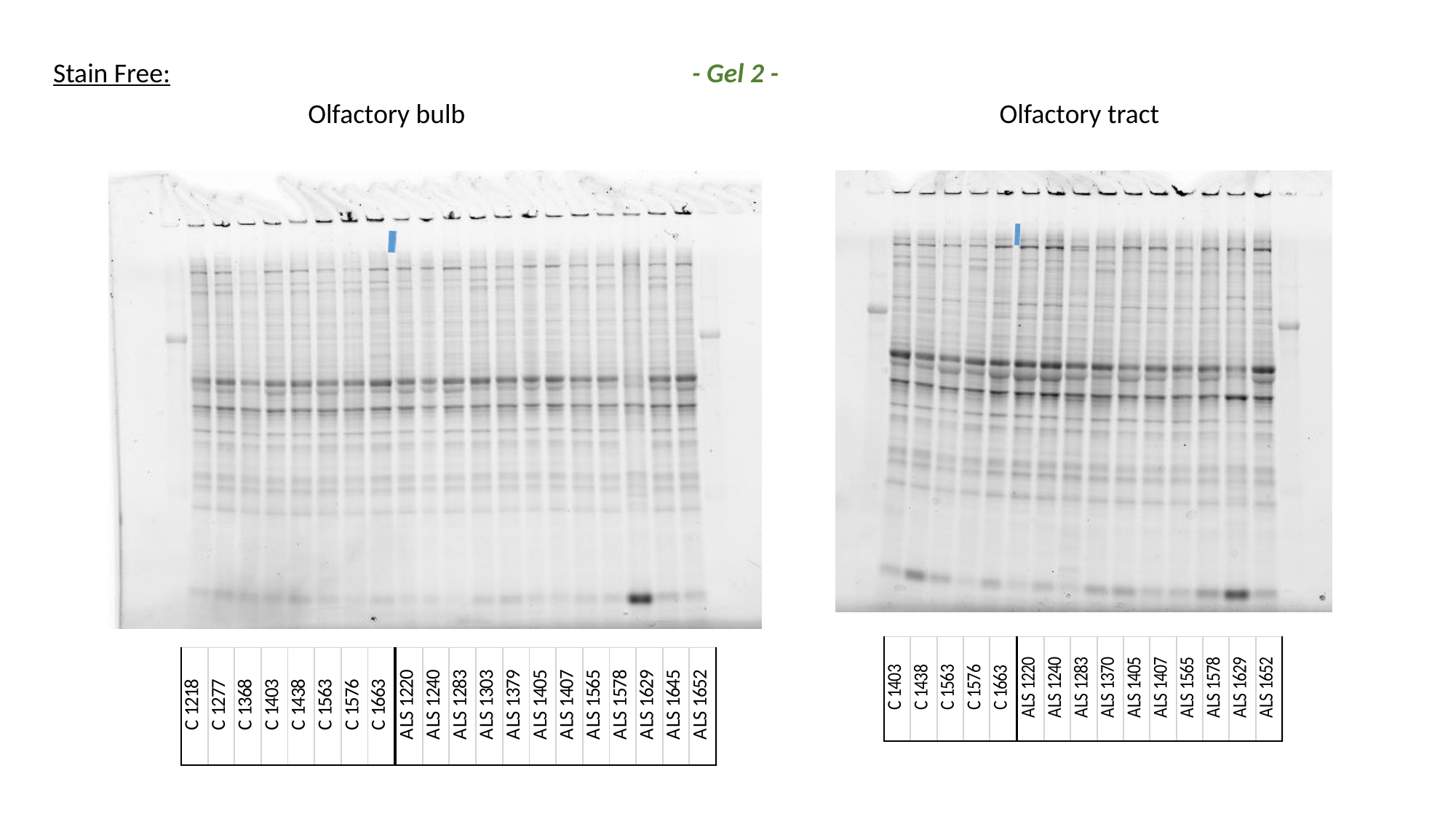

Stain Free:
- Gel 2 -
Olfactory bulb
Olfactory tract

## Slide 5
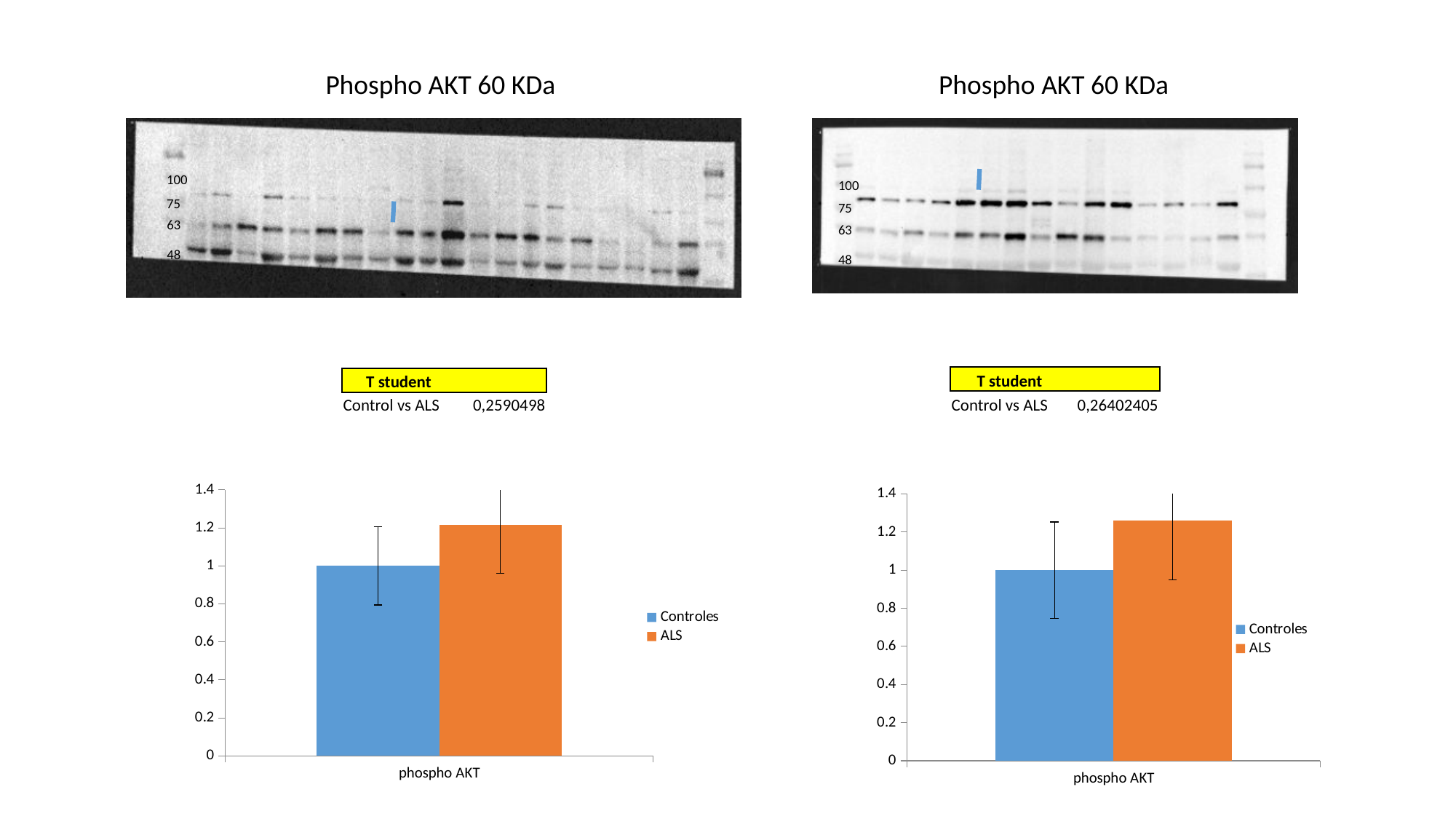

Phospho AKT 60 KDa
Phospho AKT 60 KDa
100
100
75
75
63
63
48
48
| T student | |
| --- | --- |
| Control vs ALS | 0,26402405 |
| T student | |
| --- | --- |
| Control vs ALS | 0,2590498 |
### Chart
| Category | Controles | ALS |
|---|---|---|
| phospho AKT | 0.9999999999999999 | 1.2162828563548842 |
### Chart
| Category | Controles | ALS |
|---|---|---|
| phospho AKT | 1.0 | 1.2603766463957293 |

## Slide 6
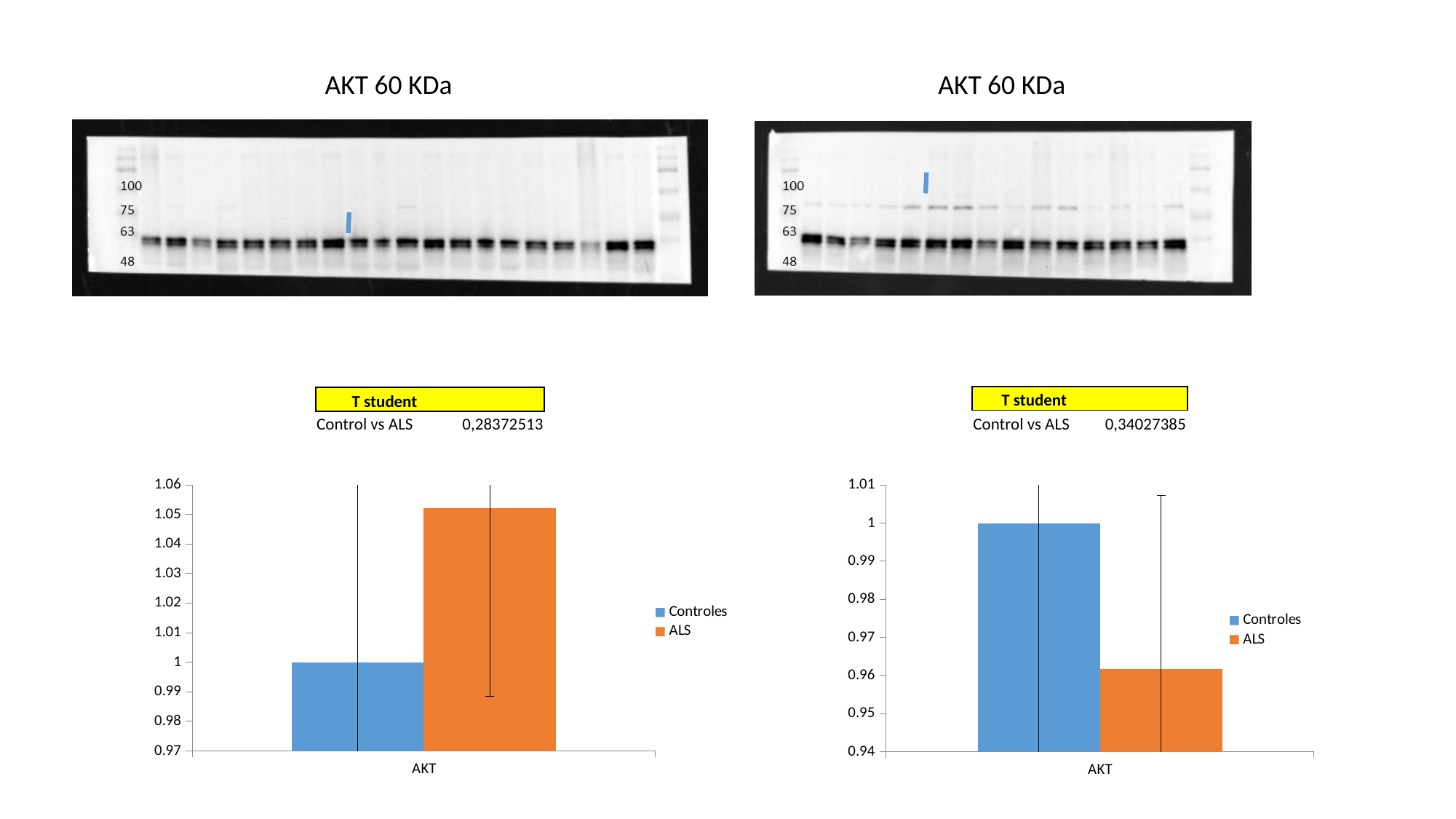

AKT 60 KDa
AKT 60 KDa
| T student | |
| --- | --- |
| Control vs ALS | 0,34027385 |
| T student | |
| --- | --- |
| Control vs ALS | 0,28372513 |
### Chart
| Category | Controles | ALS |
|---|---|---|
| AKT | 1.0000000000000002 | 1.052034059066171 |
### Chart
| Category | Controles | ALS |
|---|---|---|
| AKT | 1.0000000000000002 | 0.9616165082209172 |

## Slide 7
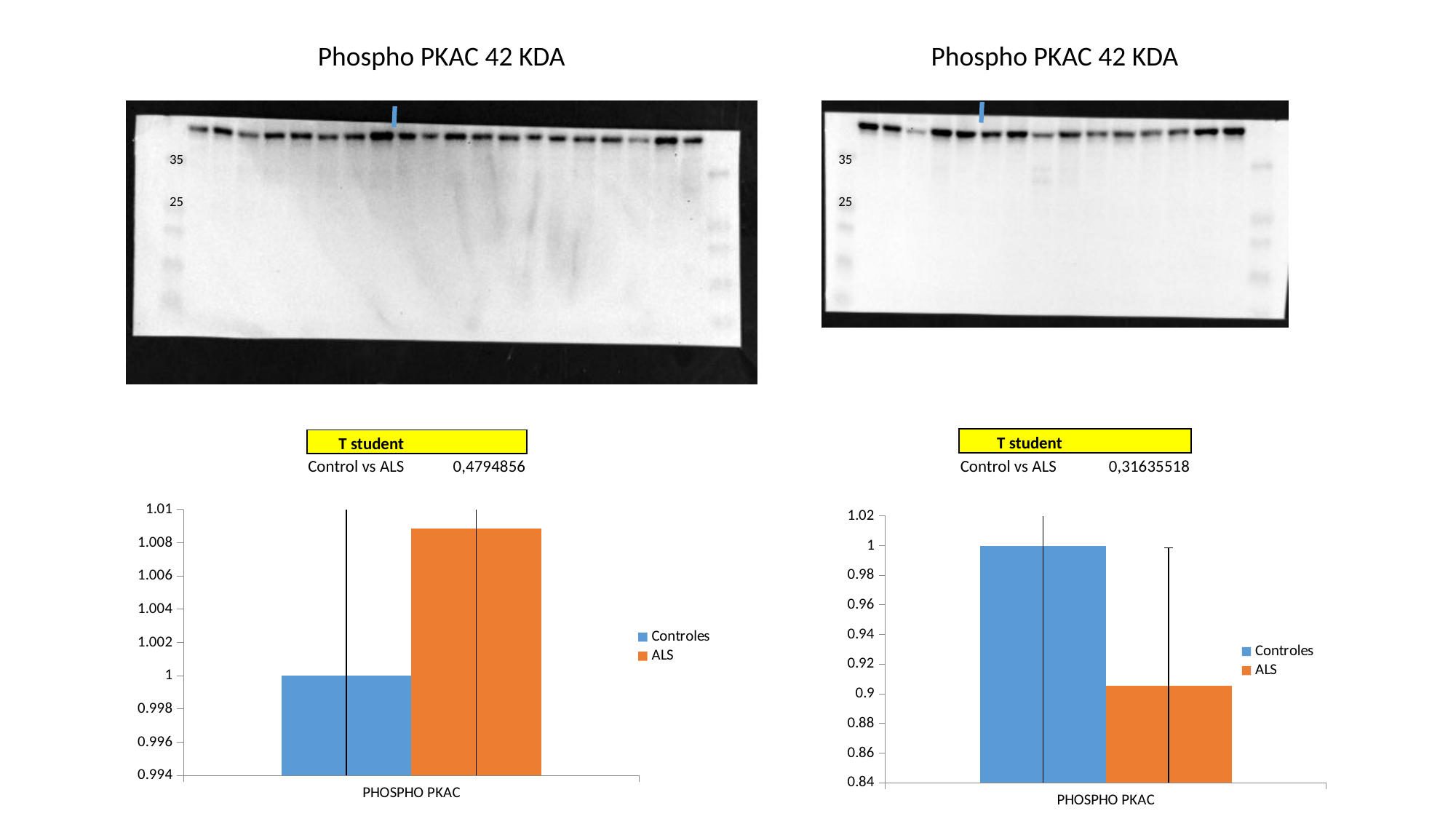

Phospho PKAC 42 KDA
Phospho PKAC 42 KDA
35
35
25
25
| T student | |
| --- | --- |
| Control vs ALS | 0,31635518 |
| T student | |
| --- | --- |
| Control vs ALS | 0,4794856 |
### Chart
| Category | Controles | ALS |
|---|---|---|
| PHOSPHO PKAC | 1.0 | 1.0088591886864882 |
### Chart
| Category | Controles | ALS |
|---|---|---|
| PHOSPHO PKAC | 1.0 | 0.9052776183907213 |

## Slide 8
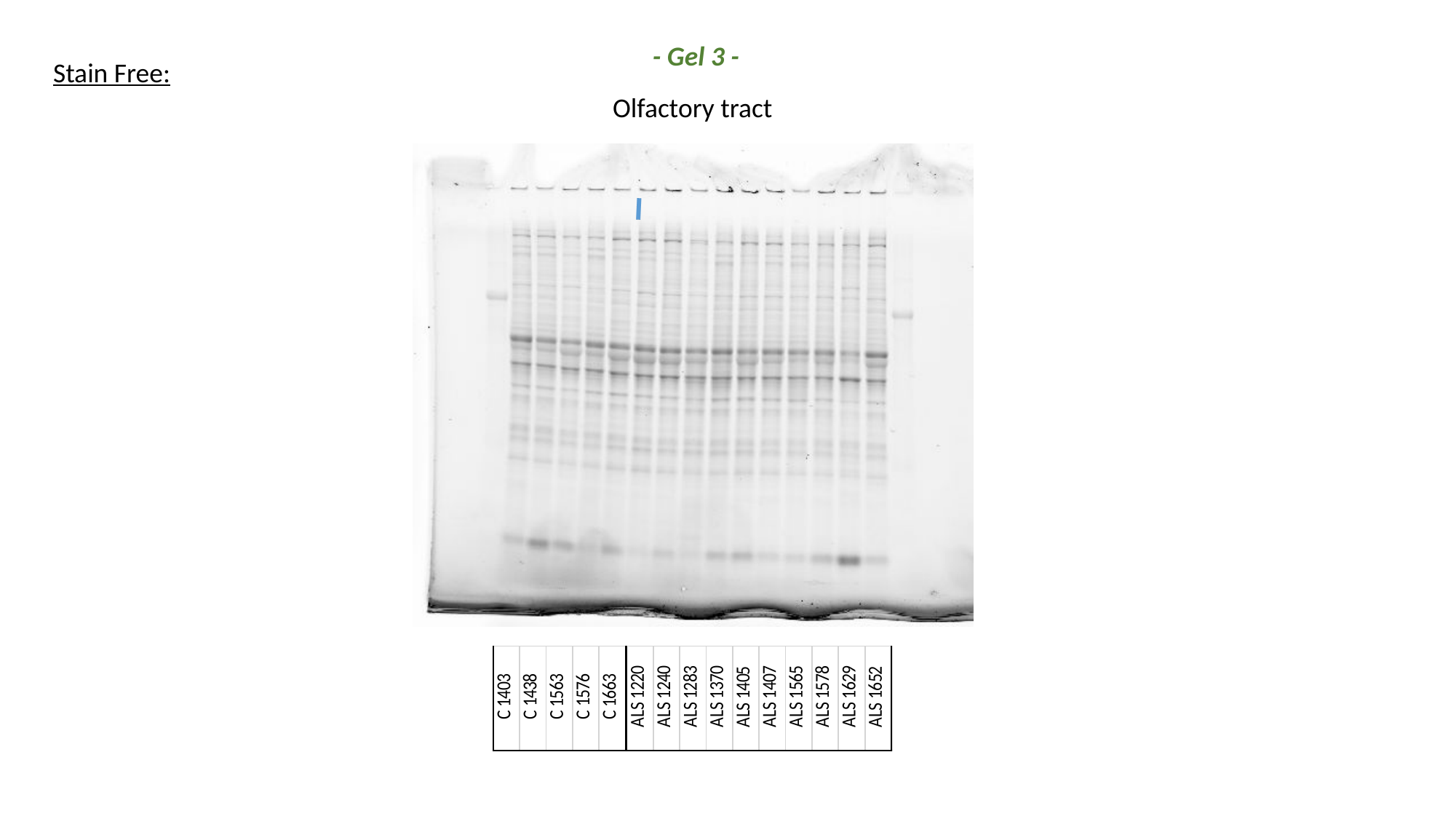

- Gel 3 -
Stain Free:
Olfactory tract

## Slide 9
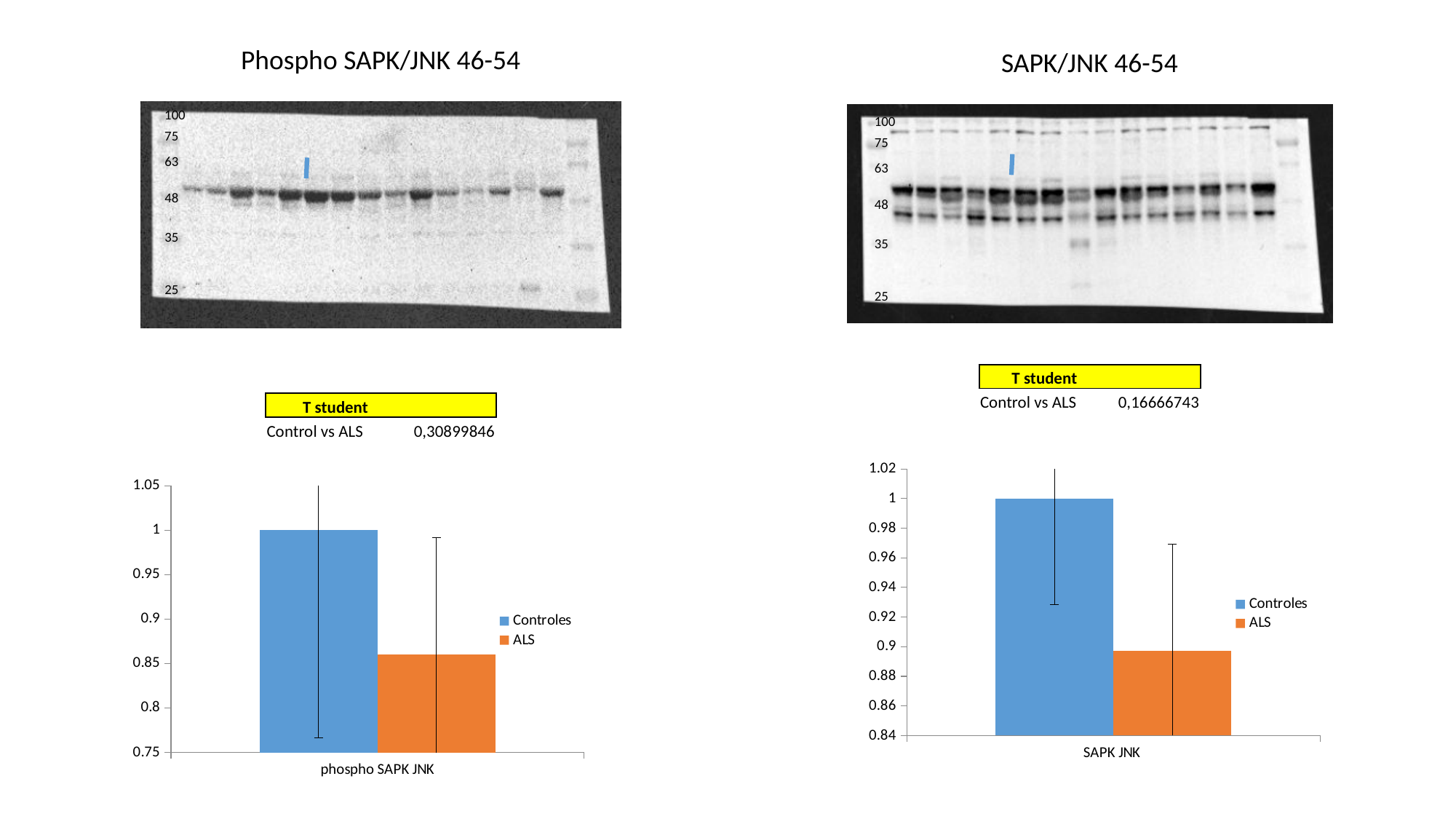

Phospho SAPK/JNK 46-54
SAPK/JNK 46-54
100
100
75
75
63
63
48
48
35
35
25
25
| T student | |
| --- | --- |
| Control vs ALS | 0,16666743 |
| T student | |
| --- | --- |
| Control vs ALS | 0,30899846 |
### Chart
| Category | Controles | ALS |
|---|---|---|
| SAPK JNK | 0.9999999999999998 | 0.8973111370212958 |
### Chart
| Category | Controles | ALS |
|---|---|---|
| phospho SAPK JNK | 1.0 | 0.8599301122915485 |

## Slide 10
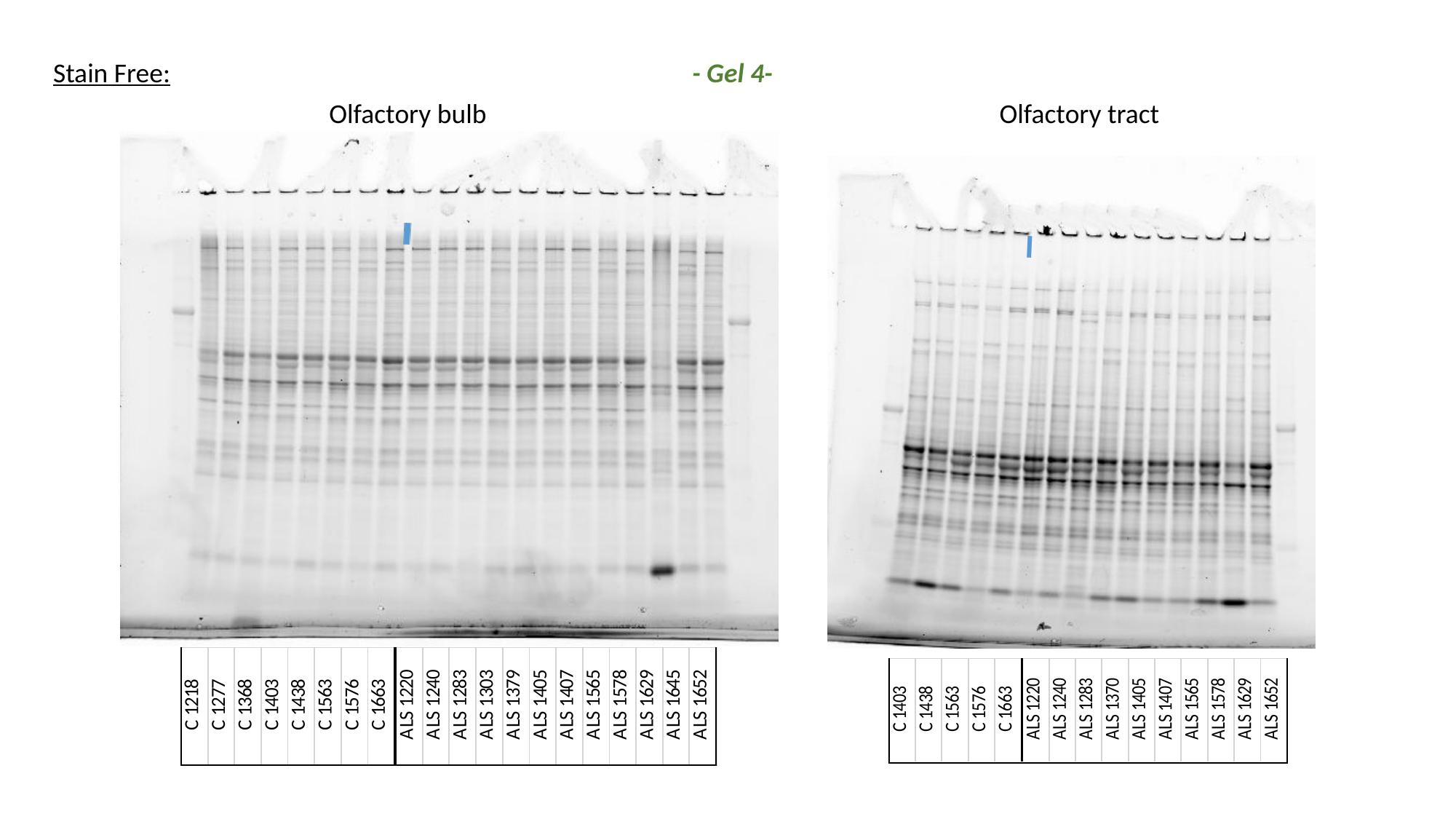

Stain Free:
- Gel 4-
Olfactory bulb
Olfactory tract

## Slide 11
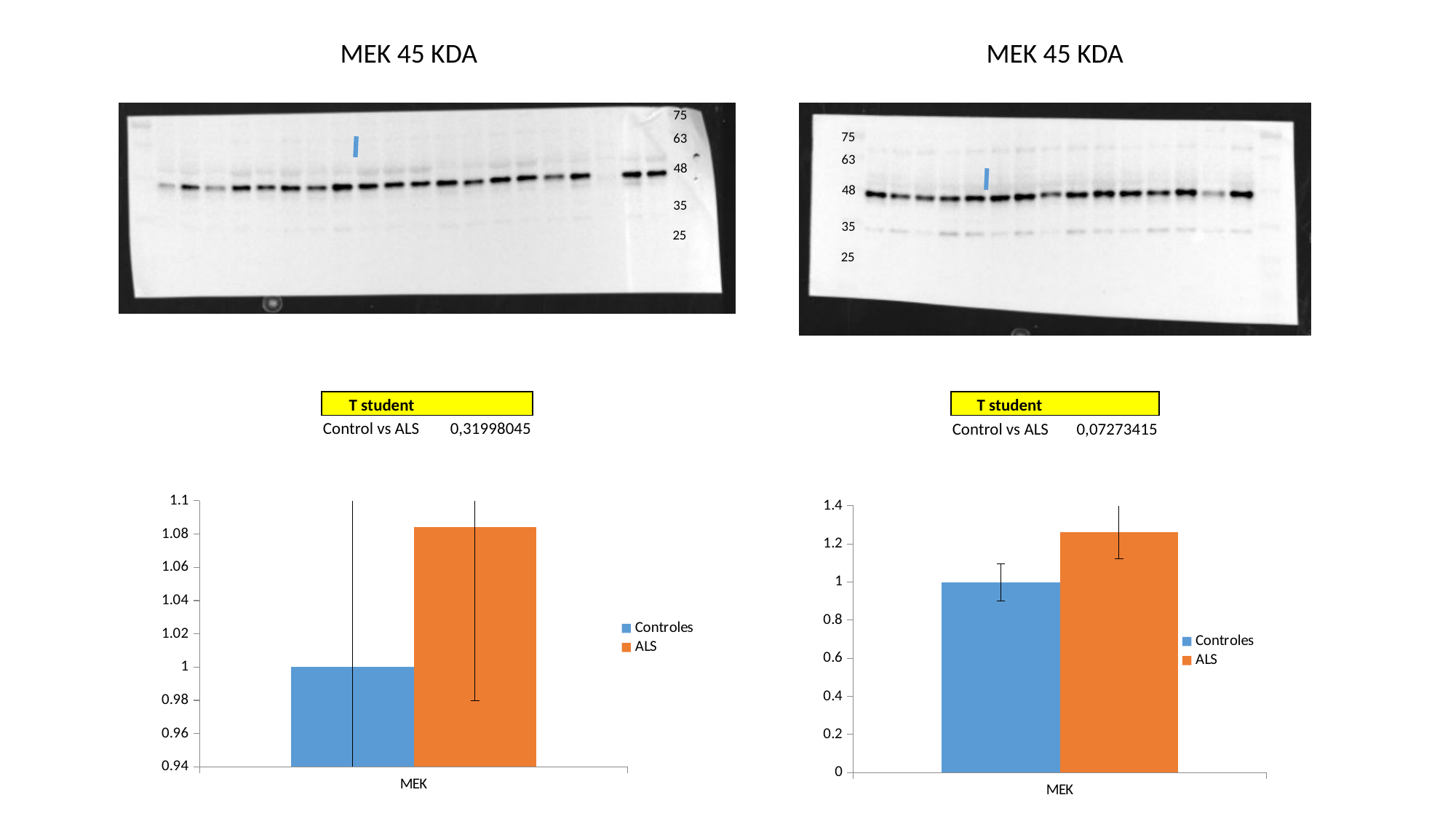

MEK 45 KDA
MEK 45 KDA
75
75
63
63
48
48
35
35
25
25
| T student | |
| --- | --- |
| Control vs ALS | 0,31998045 |
| T student | |
| --- | --- |
| Control vs ALS | 0,07273415 |
### Chart
| Category | Controles | ALS |
|---|---|---|
| MEK | 0.9999999999999999 | 1.0843257435555225 |
### Chart
| Category | Controles | ALS |
|---|---|---|
| MEK | 1.0 | 1.263665528287412 |

## Slide 12
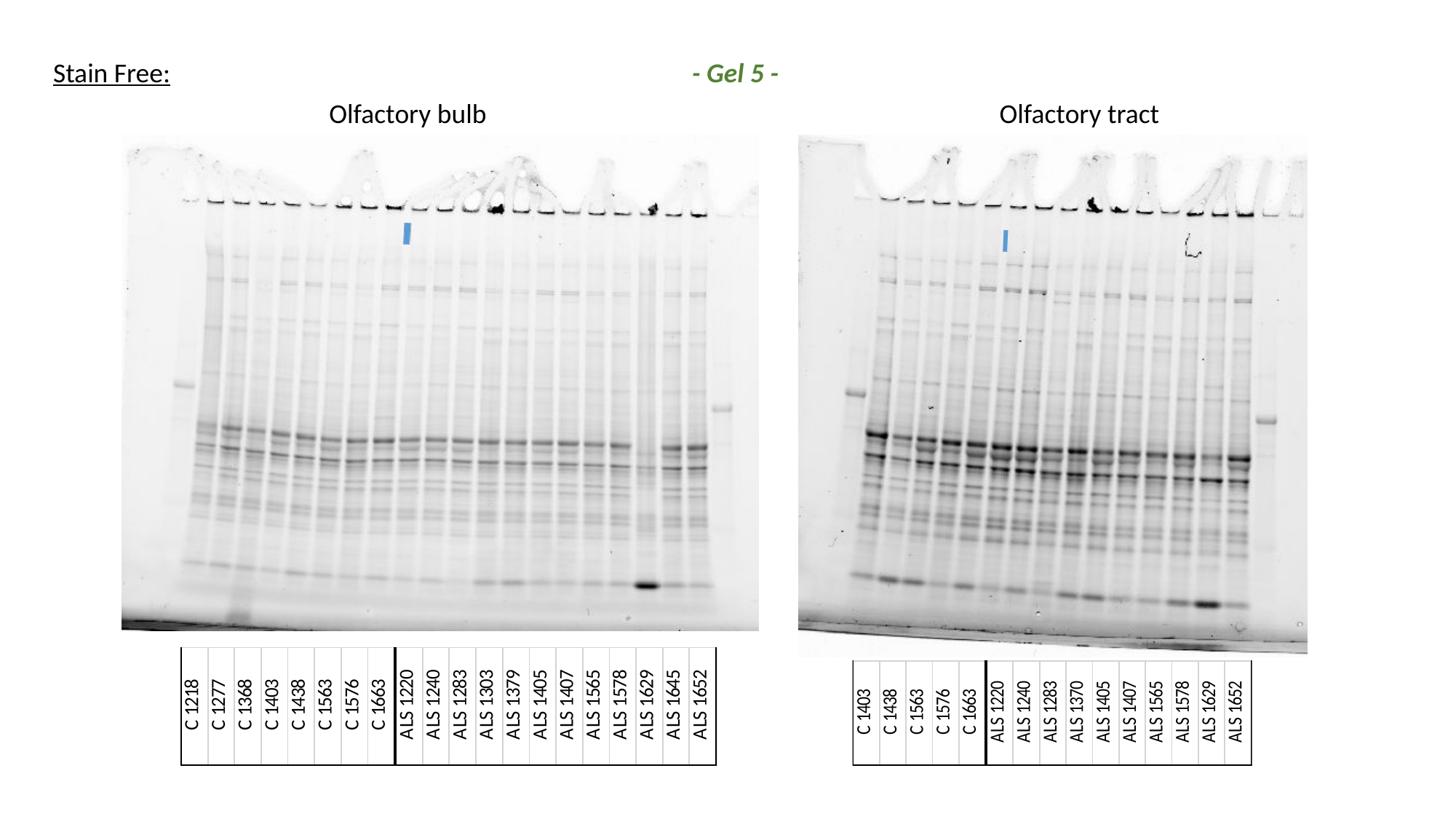

Stain Free:
- Gel 5 -
Olfactory bulb
Olfactory tract

## Slide 13
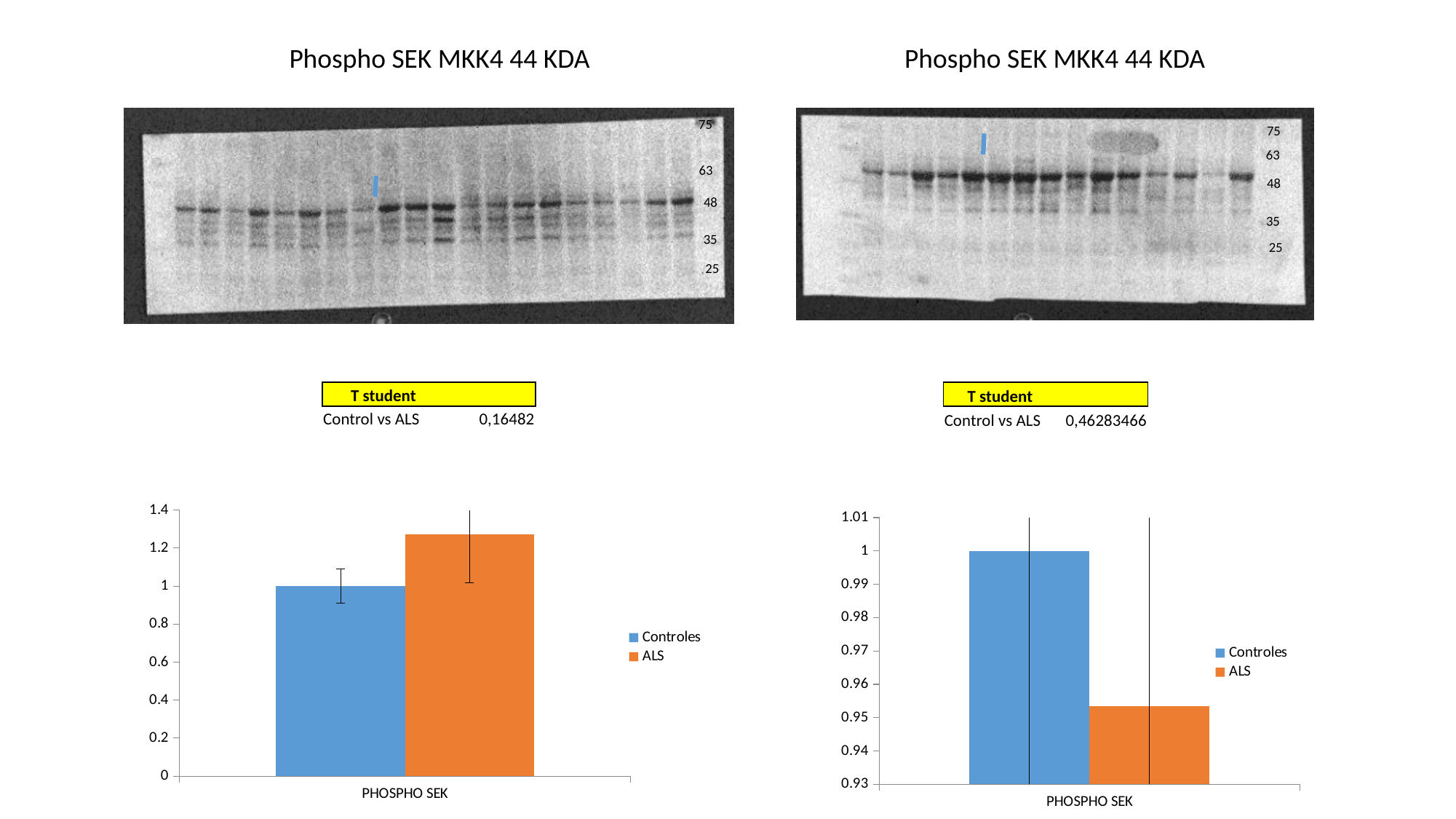

Phospho SEK MKK4 44 KDA
Phospho SEK MKK4 44 KDA
75
75
63
63
48
48
35
35
25
25
| T student | |
| --- | --- |
| Control vs ALS | 0,16482 |
| T student | |
| --- | --- |
| Control vs ALS | 0,46283466 |
### Chart
| Category | Controles | ALS |
|---|---|---|
| PHOSPHO SEK | 1.0000000000000002 | 1.272786729855453 |
### Chart
| Category | Controles | ALS |
|---|---|---|
| PHOSPHO SEK | 1.0 | 0.9533583956840911 |

## Slide 14
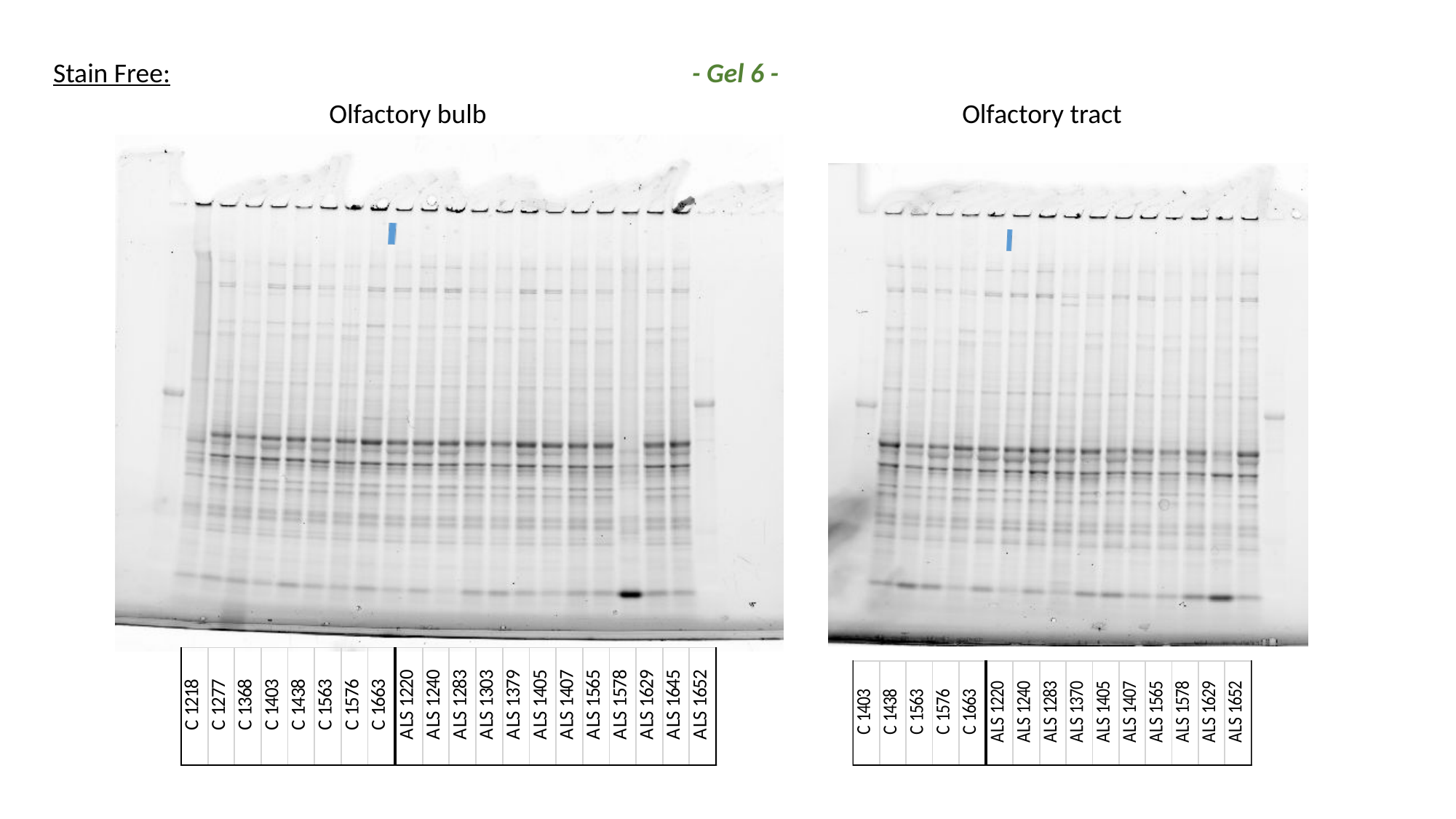

Stain Free:
- Gel 6 -
Olfactory bulb
Olfactory tract

## Slide 15
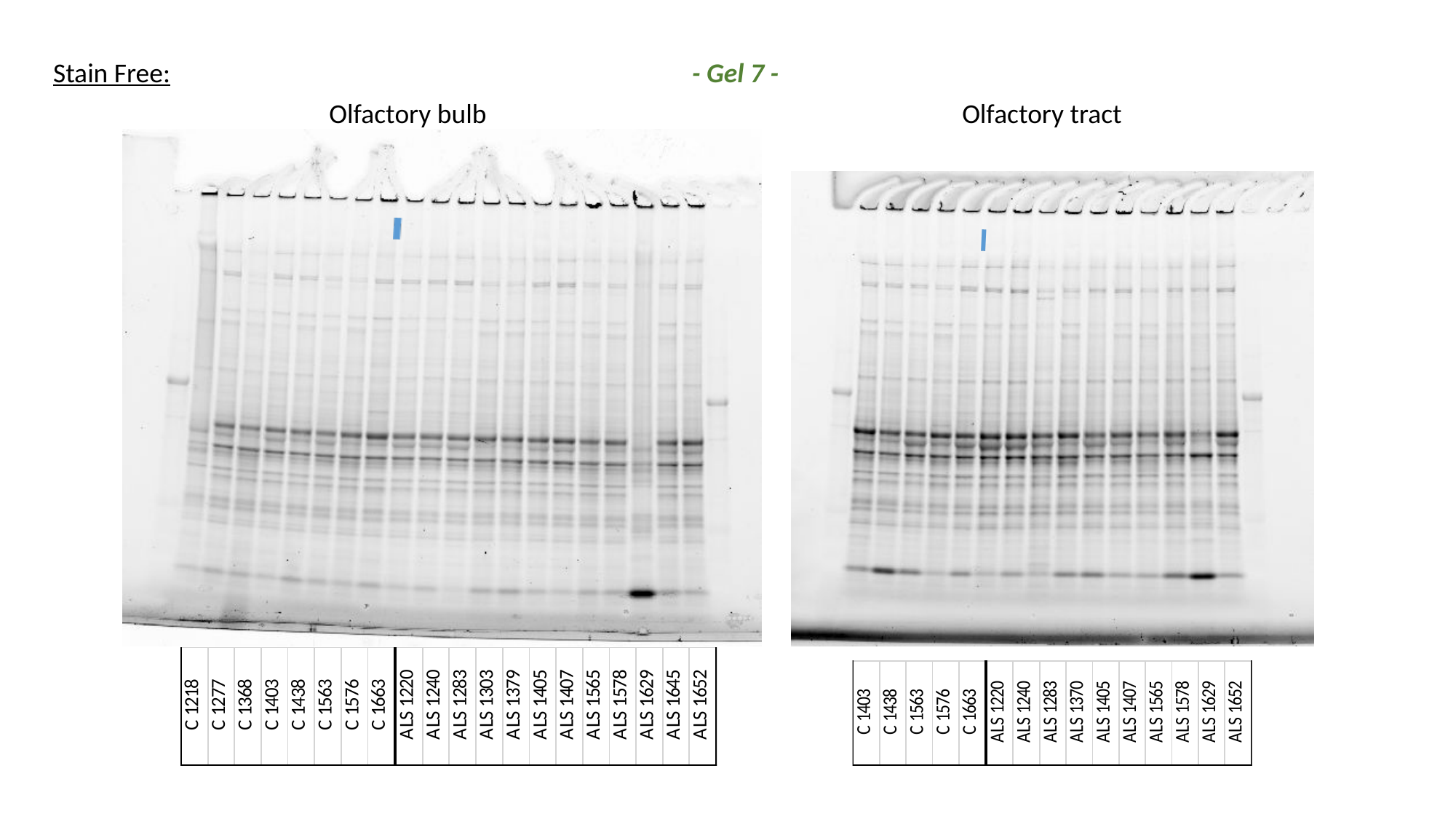

Stain Free:
- Gel 7 -
Olfactory bulb
Olfactory tract

## Slide 16
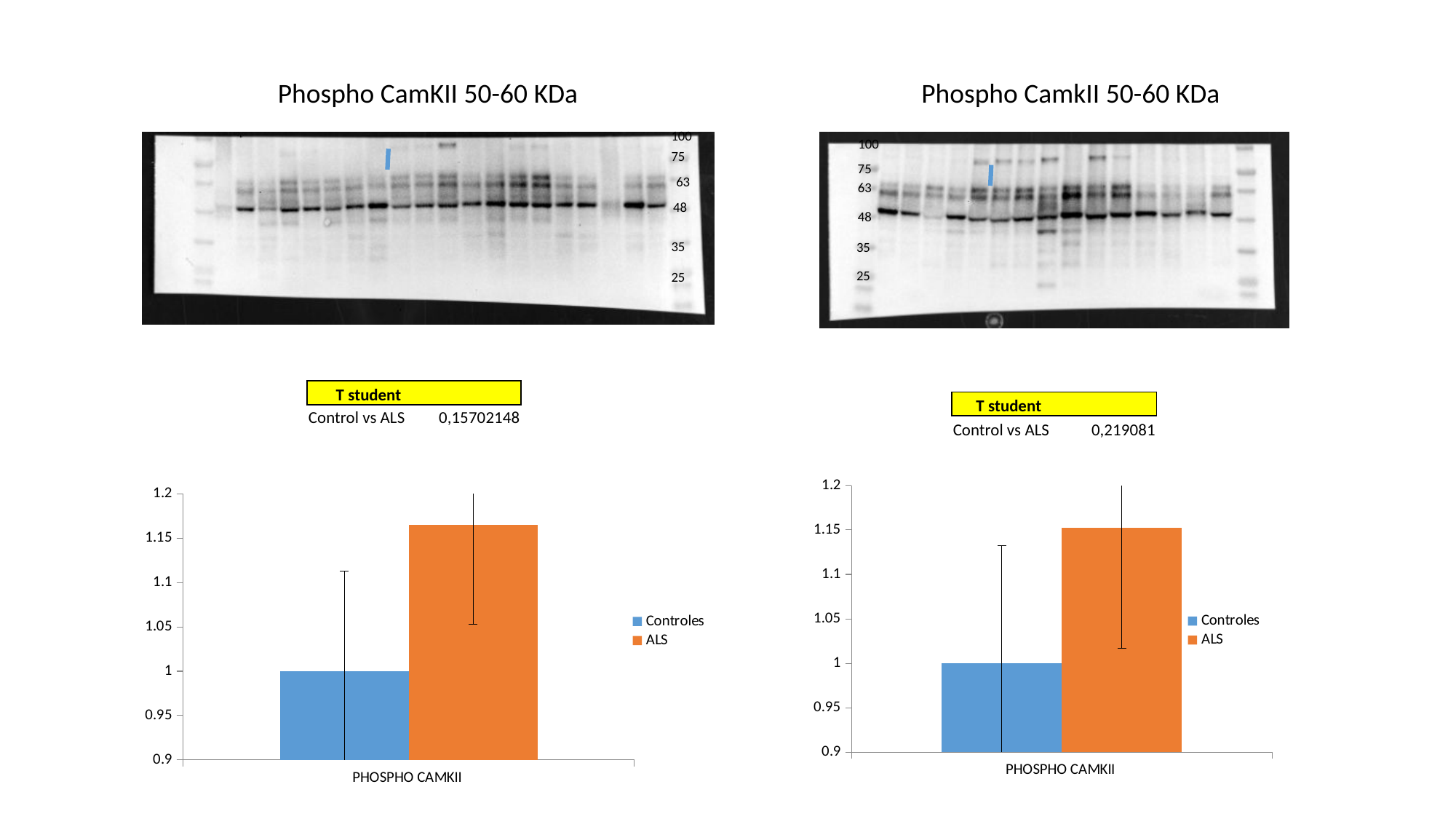

Phospho CamKII 50-60 KDa
Phospho CamkII 50-60 KDa
100
100
75
75
63
63
48
48
35
35
25
25
| T student | |
| --- | --- |
| Control vs ALS | 0,15702148 |
| T student | |
| --- | --- |
| Control vs ALS | 0,219081 |
### Chart
| Category | Controles | ALS |
|---|---|---|
| PHOSPHO CAMKII | 1.0 | 1.1522352021583526 |
### Chart
| Category | Controles | ALS |
|---|---|---|
| PHOSPHO CAMKII | 0.9999999999999999 | 1.1653385922160475 |

## Slide 17
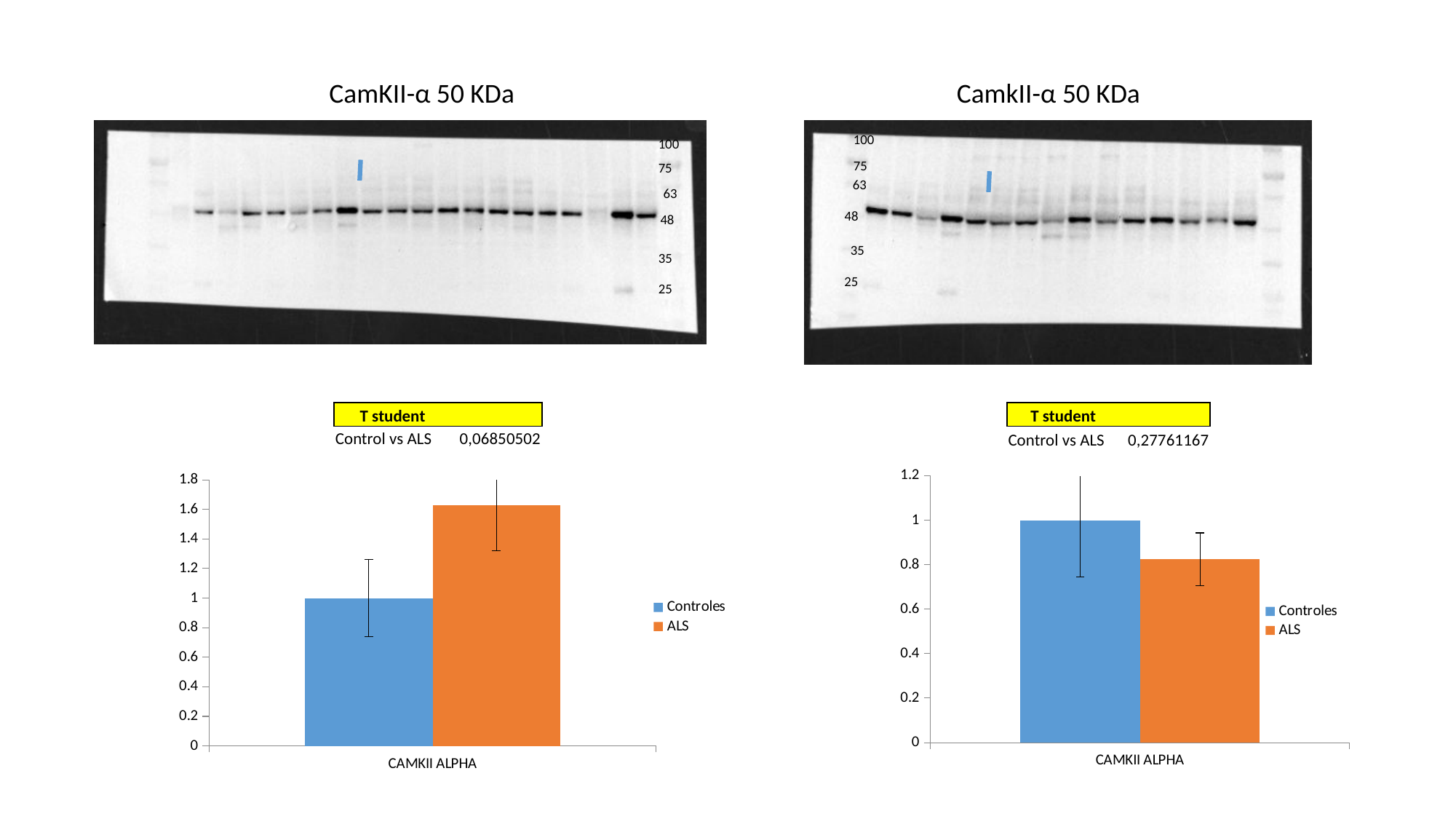

CamKII-α 50 KDa
CamkII-α 50 KDa
100
100
75
75
63
63
48
48
35
35
25
25
| T student | |
| --- | --- |
| Control vs ALS | 0,06850502 |
| T student | |
| --- | --- |
| Control vs ALS | 0,27761167 |
### Chart
| Category | Controles | ALS |
|---|---|---|
| CAMKII ALPHA | 1.0 | 1.6279624631360565 |
### Chart
| Category | Controles | ALS |
|---|---|---|
| CAMKII ALPHA | 0.9999999999999998 | 0.8251031570427049 |
